# Supplementary material for: Changes in the stool and oropharyngeal microbiome in obsessive-compulsive disorder
Source: Sci Rep. 2022 Jan 27;12:1448. doi: 10.1038/s41598-022-05480-9 (PMC8795436; doi:10.1038/s41598-022-05480-9)
Supplement: Supplementary file 1 — Supplementary Information. [file 41598_2022_5480_MOESM1_ESM.pdf]

Changes in the stool and oropharyngeal microbiome in obsessive-compulsive disorder

Supplementary methods:

Diversity measures

We estimated α- and β-diversity measures within samples using the Phyloseq, picante (version 1.6.2) and vegan (version 2.4.6) R packages. α-diversity refers to species richness (number of taxa) within a single sample, while β-diversity refers to dissimilarity in taxonomic abundance profiles from different samples.
We estimated α-diversity using different indices, which give slightly different information. These include the Observed diversity, Chao1 index, Abundance-based Coverage Estimator (ACE), Shannon, Simpson, Inverse Simpson, and Fisher Diversity indices using the estimate_richness function from the Phyloseq package. We also calculated Faith's phylogenetic diversity and species richness using the pd function from the picante package (version 1.6.2). Boxplots were generated using ggplot2 (version 2.2.1). Statistical significance of α-diversity differences between groups was evaluated with Mann–Whitney U test when samples were independent, and with Wilcoxon rank-sum test when samples were paired.
The observed diversity index measures the number of different species per sample, which is defined as "richness". It does not consider the abundances of the species or their relative abundance distributions. The Chao1 index is also a qualitatively measure of alpha diversity which, beside species richness, considers the ratio of singletons (n = 1) to doubletons (n = 2) giving more weight to rare species. The ACE incorporates data from all species with fewer than 10 individuals, rather than just singletons and doubletons. The Shannon diversity index relates taxa richness and evenness, which is defined as the

relative abundances of the different species making up the samples' richness. The
Simpson diversity index considers the number of species present, as well as the abundance of each species, but it has a strong dependency on the few most common species. The inverse of Simpson index refers to the effective number of taxa types that is obtained when the weighted arithmetic mean is used to quantify average proportional abundance of taxa types in the dataset of interest. Fisher is an alpha diversity measure with an inherent assumption of a logarithmic series-type rank abundance structure of communities. Finally, Faith's Phylogenetic Diversity (PD) is the phylogenetic analogue of taxon richness and is expressed as the number of tree units which are found in a sample.

We estimated β-diversity as the weighted and unweighted UniFrac distance between samples with the Unifrac function, as well as the Jensen-Shannon Divergence (JSD) with the JSD function, both from the Phyloseq package. We also calculated the Bray-Curtis dissimilarity and Canberra index using the vegdist function in the vegan package (version 2.4.6). Furthermore, the adonis function in the vegan package was used to perform a PERMANOVA test on β-diversity with 999 permutations considering even dependence of samples (paired OCD samples after and before treatment) using the "strata" argument within the adonis function. We used a Principal Coordinate Analysis (PCoA) to visualize the clustering of the samples.
Bray-Curtis dissimilarity is a statistic used to quantify the compositional dissimilarity between two samples, based on abundance or read count data. It is dominated by the abundant species so that rare species add very little to the value of the coefficient. The Canberra metric is not affected as much by the more abundant species in the community, and thus differs from the Bray-Curtis

measure. The Jensen–Shannon divergence is a method of measuring the similarity between two probability distributions. UniFrac is a distance metric used for comparing biological communities. It differs from dissimilarity measures in that it incorporates information on the relative relatedness of community members by incorporating phylogenetic distances between observed organisms in the computation. Unweighted uniFrac metric is purely based on sequence distances (does not include abundance information), while in weighted UniFrac metric branch lengths are weighted by relative abundances (includes both sequence and abundance information).

Supplementary figures

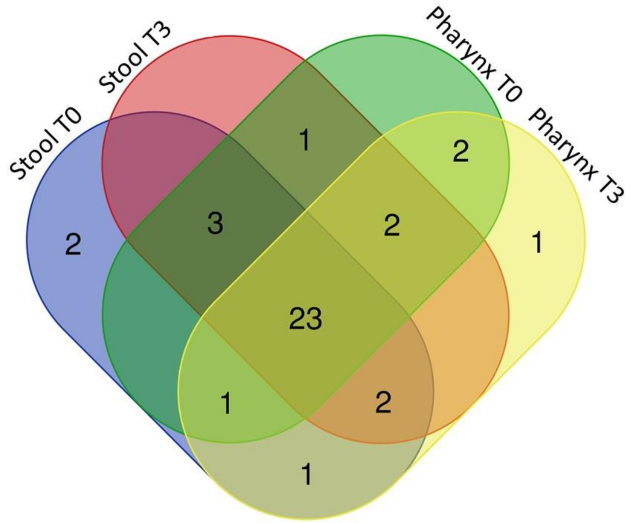

Figure S1. Venn diagram representing the overlap between all collected OCD stool and pharynxs samples. 38 total individuals were sampled. For 23 of them we were able to obtain DNA from all types of samples, while for the remaining 15 DNA was only obtained for 1, 2 or 3 of the different sample types, as detailed by the overlapping circles in the venn diagram.

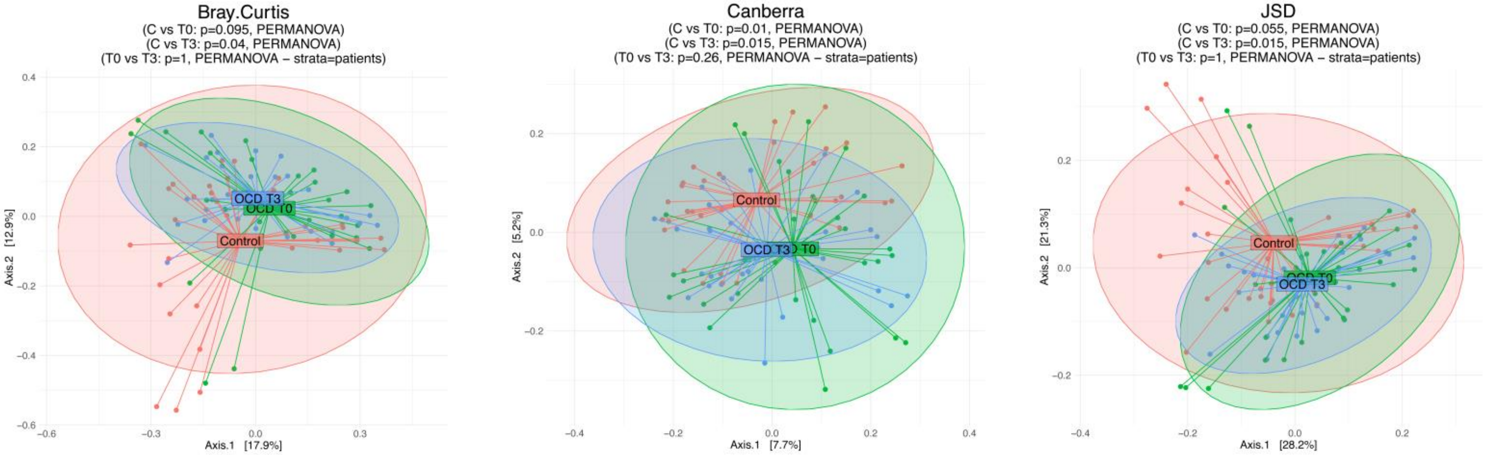

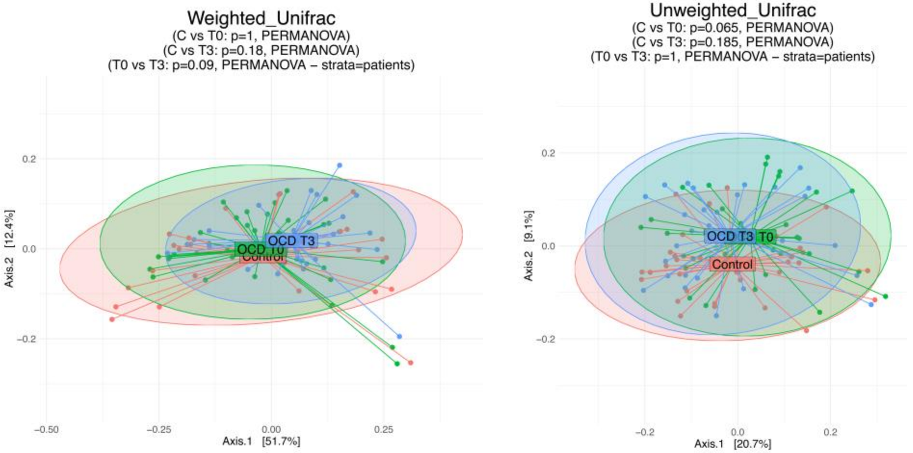

Figure S2. Principal coordinate analysis plot of OCD T0 (green), OCD T3 (blue) and control (red) groups in stool samples. The plots show the two principal coordinates for principal coordinates analysis (PCoA) using Bray-Curtis, Canberra, Jensen-Shannon, unweighted UniFrac and weighted UniFrac algorithms. The resulting p-values for PERMANOVA analyses are reported iAn each plot.

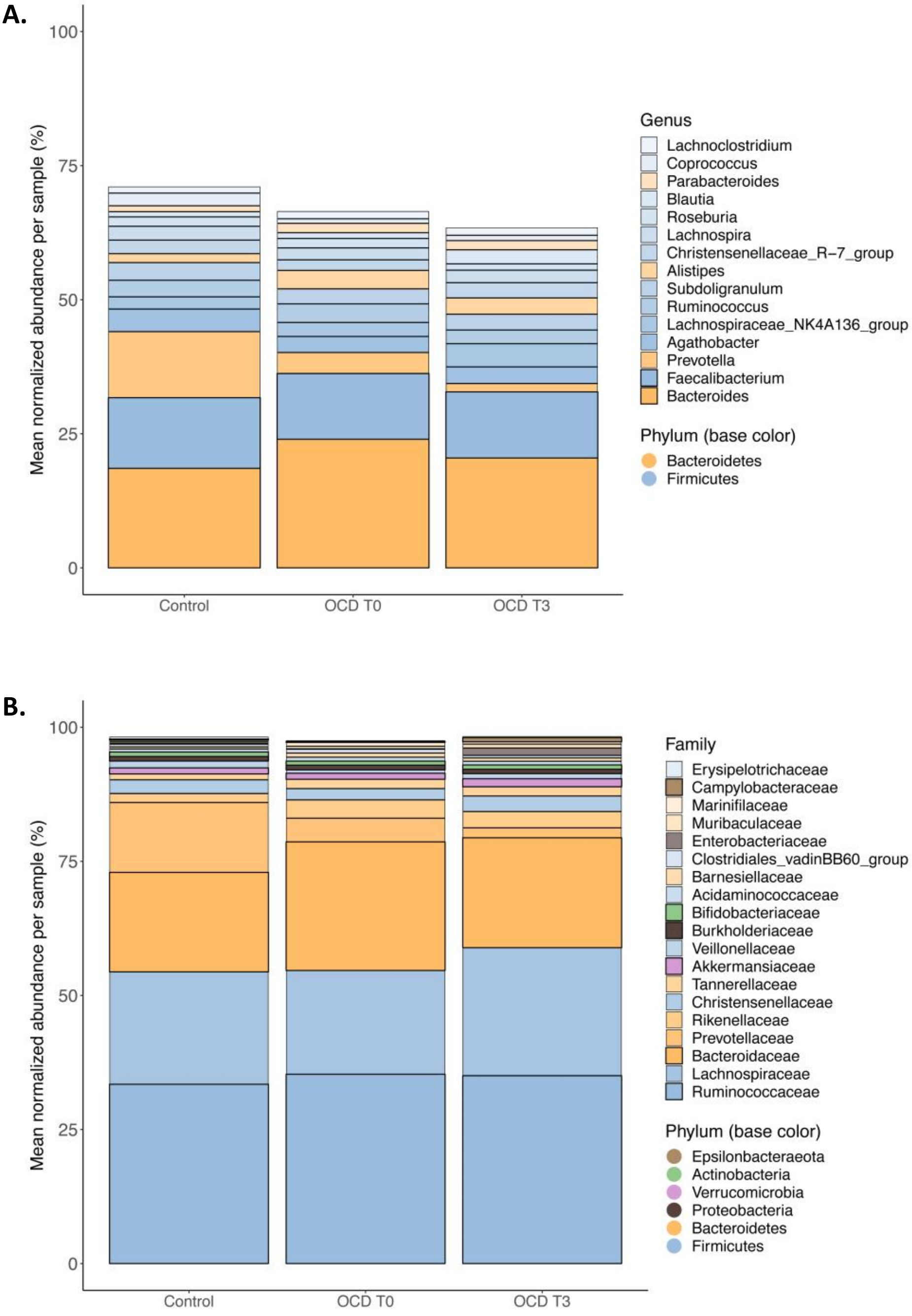

Figure S3. Stool bacterial abundances for OCD T0, OCD T3 and controls at the A. genus and B. family level.

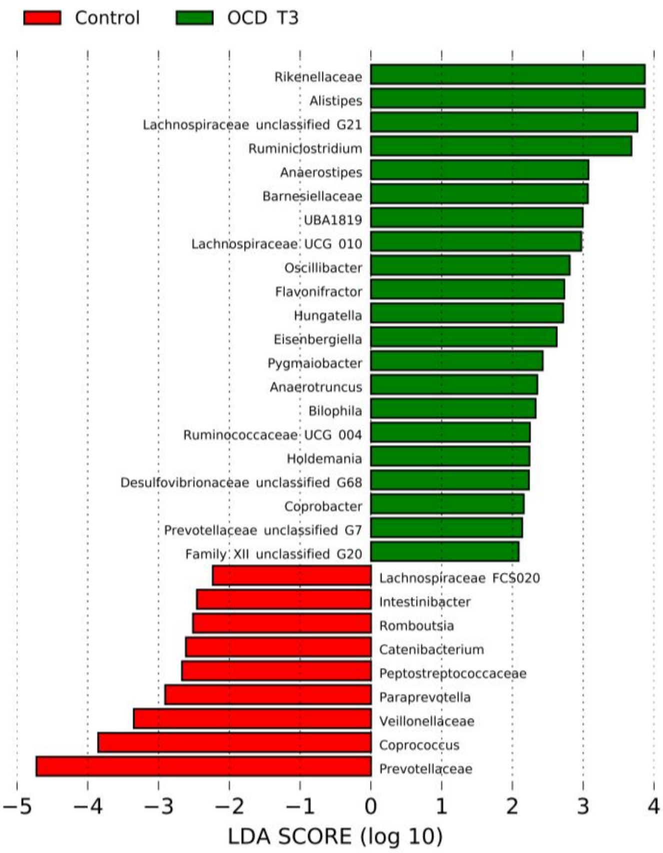

Figure S4. Biomarkers associated with OCD T3 and control groups discovered by a linear discriminant effect size (LEfSe) analysis (α value=0.05, logarithmic LDA score threshold=2.0) in stool.

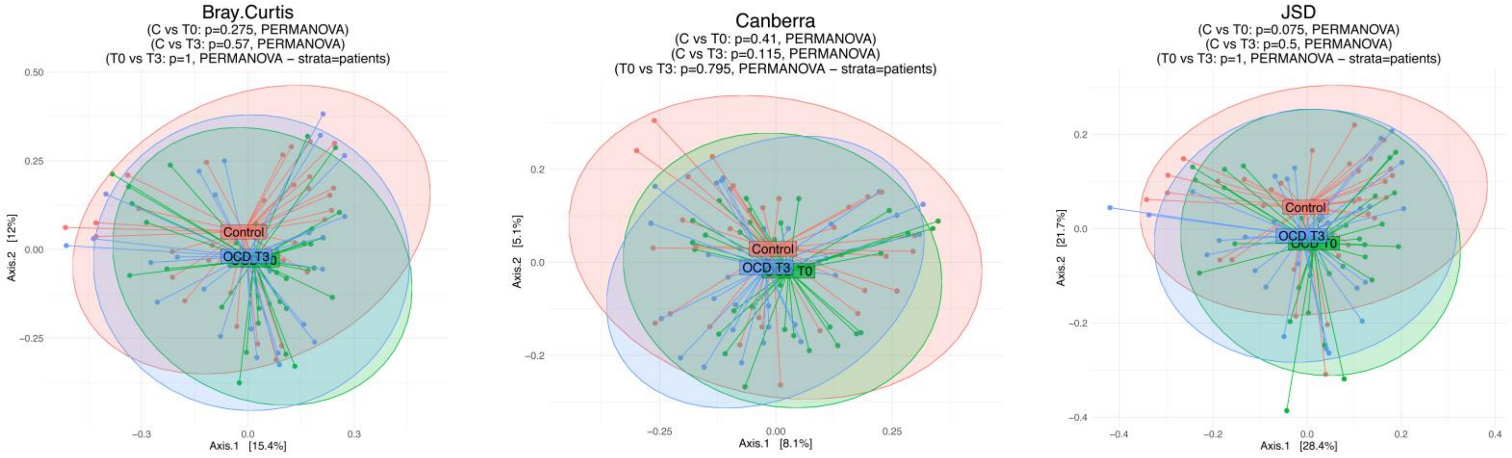

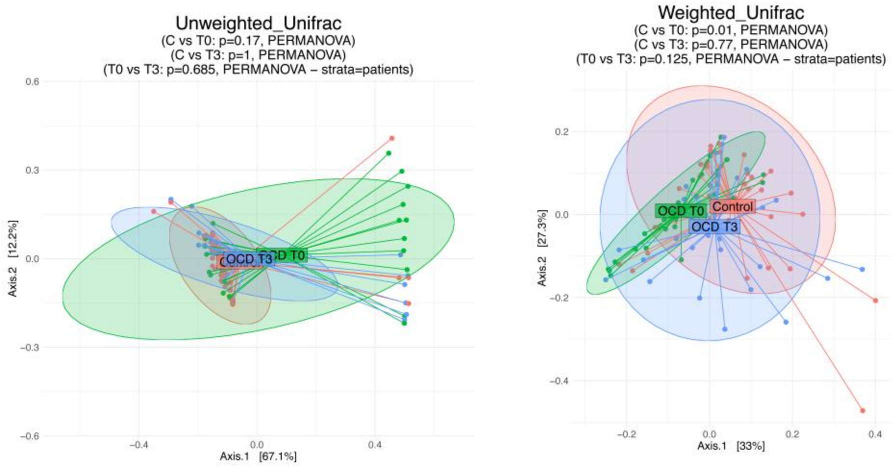

Figure S5. Principal coordinate analysis plot of OCD T0 (green), OCD T3 (blue) and control (red) groups in oropharyngeal samples. The plots show the two principal coordinates for principal coordinates analysis (PCoA) using Bray-Curtis, Canberra, Jensen-Shannon, unweighted UniFrac and weighted UniFrac algorithms. The resulting p-values for PERMANOVA analyses are reported in each plot.

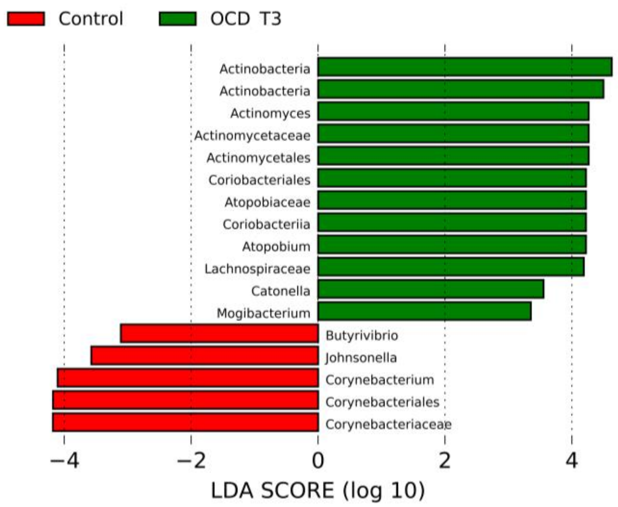

Figure S6. Biomarkers associated with OCD T3 and control groups discovered by a linear discriminant effect size (LEfSe) analysis (α value=0.05, logarithmic LDA score threshold=2.0) in oropharyngeal samples.

A.

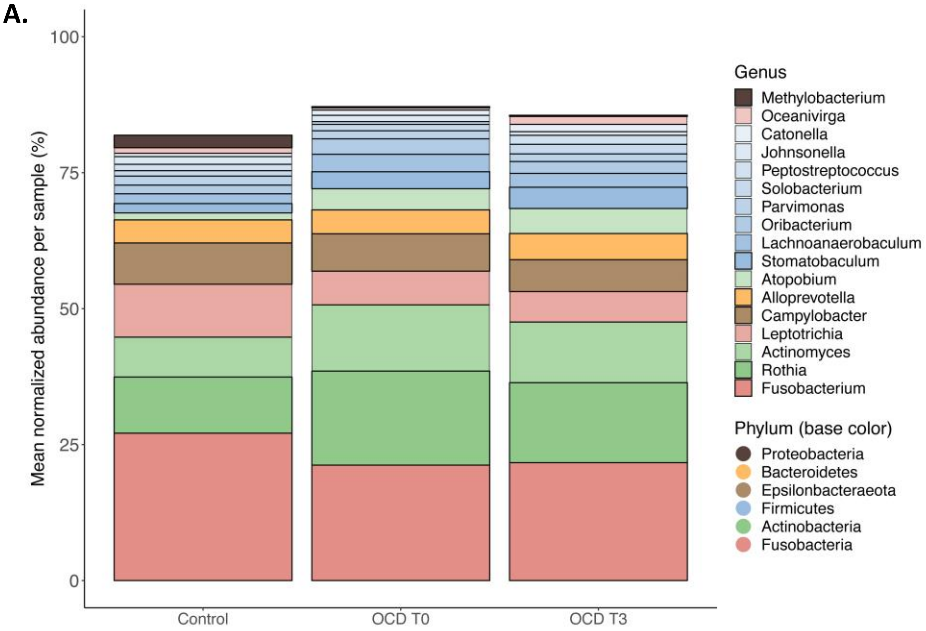

B.

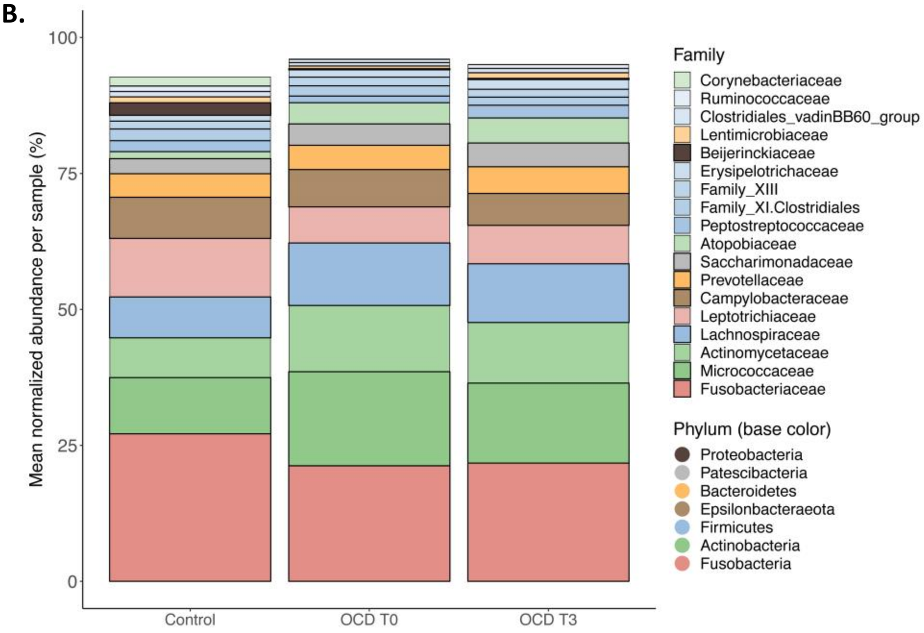

Figure S7. Oropharyngeal bacterial abundances for OCD T0, OCD T3 and controls at the A. genus and B. family level.

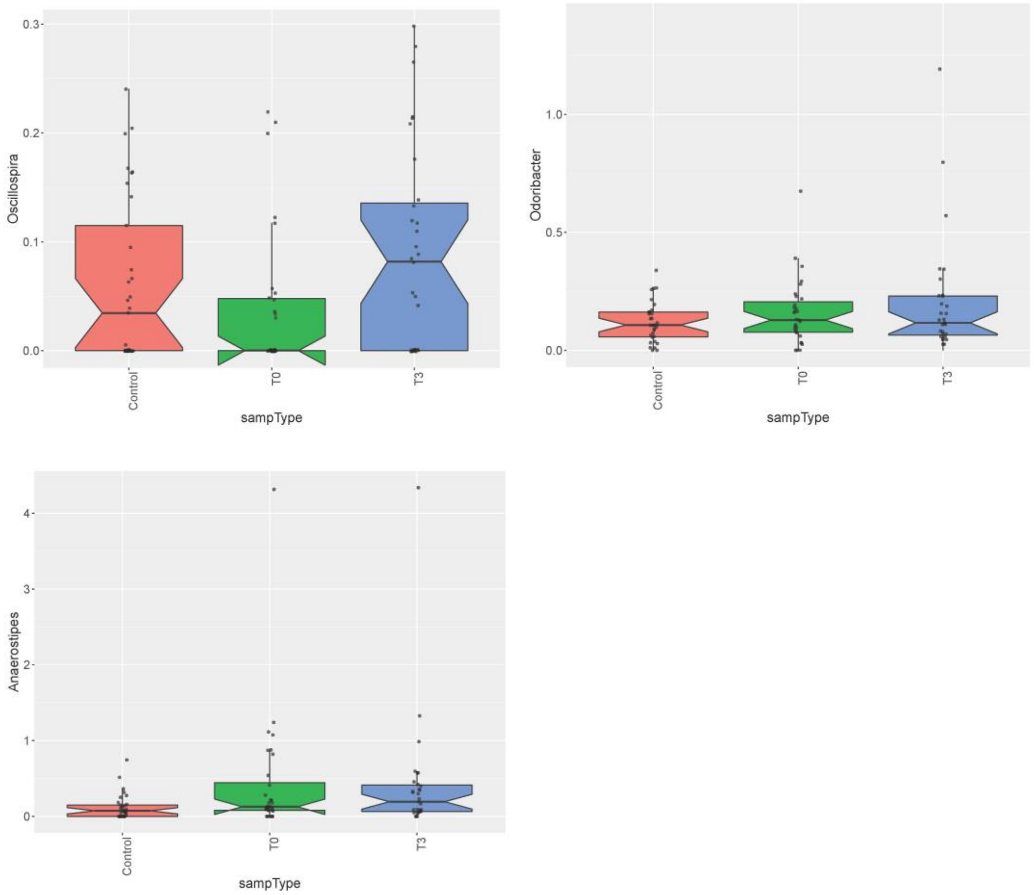

Figure S8. Box plot representation of the relative abundances in stool for the genera Odoribacter, Oscillospira, and Anaerostipes, in the three groups of samples, OCD T0, OCD T3 and Contol. One extreme outlier with a value of 12.55 in OCD T0 was removed from the graph for Odoribacter to ease data visualization. The relative abundance of Anaerostipes in OCD T0 and OCD T3 are both significantly different from the Control group.

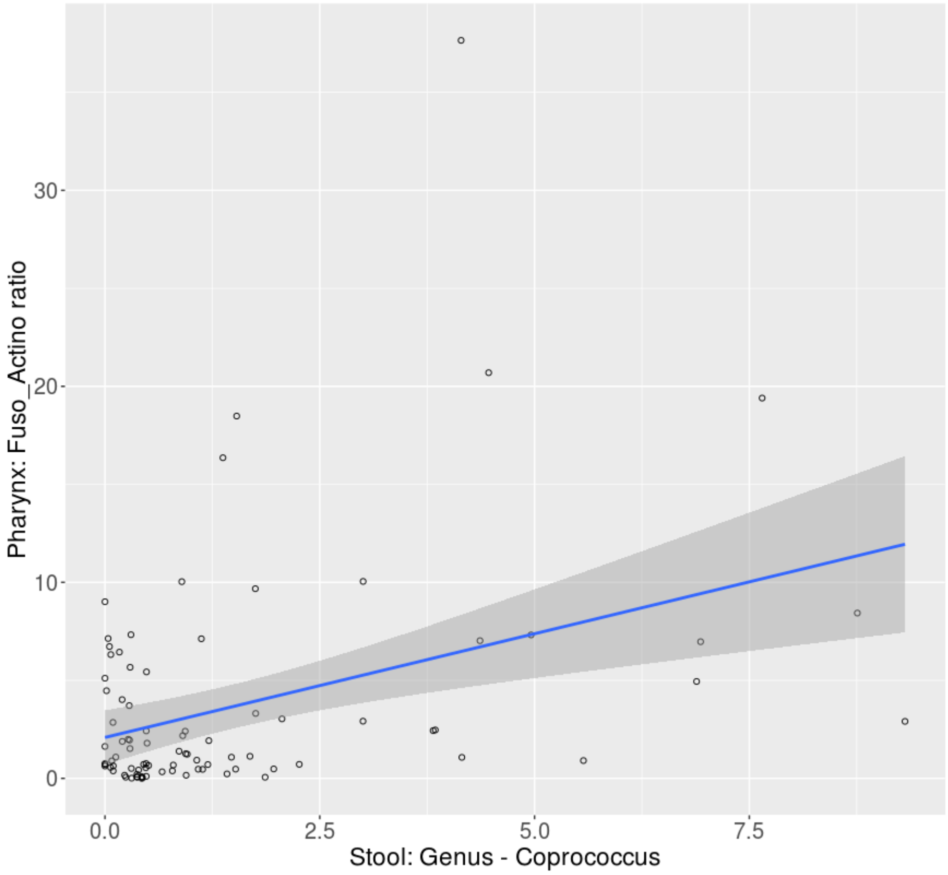

Figure S9. Plot of the correlation (within individuals) of features in pharynx and stool: correlation between the fusobacteria/actinobacteria ratio in pharynx and the presence of Coprococcus in stool samples. correlation coefficient = 0.379, adjusted p-val = 0.0378

Supplementary Table 1. Clinical data from OCD samples

Sample	Stool sample T0	Stool sample T3	Pharyngeal swab sample T0	Pharynx swab sample T3	Gender	Age	Y-BOCS T0	Y-BOCS Obs T0	Y-BOCS Comp T0	HRDS T0	Y-BOCS T3	-BOCS Obs T	BOCS Comp	Improveme	Response
OCD 2026	✓	✓	✓	✓	2	21	18	9	9	7	14	7	7	0.22	0
OCD 1011	✓	✗	✗	✗	1	39	20	10	10	9	13	7	6	0.35	1
OCD 2024	✓	✓	✓	✓	2	58	20	10	10	9	16	8	8	0.20	0
OCD 1000	✓	✓	✓	✓	1	26	22	12	10	17	19	10	9	0.14	0
OCD 953	✗	✓	✓	✓	1	36	24	14	10	10	19	11	8	0.21	0
OCD 875	✓	✓	✓	✓	2	53	25	12	13	12	18	9	9	0.28	0
OCD 644	✓	✓	✓	✓	1	57	26	13	13	10	16	8	8	0.38	1
OCD 898	✓	✓	✓	✓	2	18	26	13	13	15	20	10	10	0.23	0
OCD 1012	✓	✓	✓	✓	1	71	26	13	13	11	12	6	6	0.54	1
OCD 2030	✗	✓	✓	✓	2	42	26	13	13	13	20	10	10	0.23	0
OCD 2031	✓	✓	✓	✗	2	21	26	12	14	9	26	12	14	0.00	0
OCD 885	✓	✓	✓	✓	2	48	27	15	12	10	27	15	12	0.00	0
OCD 1031	✓	✓	✓	✓	1	28	27	14	13	14	19	10	9	0.30	0
OCD 493	✗	✗	✓	✗	2	51	28	15	13	12	28	15	13	0.00	0
OCD 834	✓	✓	✓	✓	2	54	28	15	13	17	28	15	13	0.00	0
OCD 897	✓	✓	✓	✓	2	58	28	14	14	11	24	12	12	0.14	0
OCD 1002	✓	✓	✓	✓	1	41	28	15	13	20	24	14	10	0.14	0
OCD 1014	✓	✓	✓	✓	1	24	29	15	14	14	22	11	11	0.24	0
OCD 1025	✓	✓	✗	✓	1	20	29	16	13	15	19	10	9	0.34	0
OCD 1027	✓	✓	✓	✓	1	40	29	15	14	12	21	11	10	0.28	0
OCD 893	✗	✓	✗	✓	2	46	30	15	15	12	30	15	15	0.00	0
OCD 896	✓	✗	✓	✗	2	45	30	15	15	13	18	9	9	0.40	1
OCD 899	✓	✓	✗	✓	2	19	30	15	15	12	20	10	10	0.33	0
OCD 972	✓	✓	✓	✓	1	51	30	15	15	12	24	12	12	0.20	0
OCD 998	✓	✓	✓	✓	1	53	30	15	15	18	18	9	9	0.40	1
OCD 1030	✓	✓	✓	✓	1	39	30	15	15	17	26	13	13	0.13	0
OCD 2020	✓	✓	✗	✓	2	34	30	15	15	17	26	13	13	0.13	0
OCD 2027	✓	✓	✓	✓	2	32	30	15	15	12	24	12	12	0.20	0
OCD 2029	✓	✓	✓	✓	2	20	30	15	15	14	16	8	8	0.47	1
OCD 2017	✓	✓	✓	✓	2	33	31	15	16	12	14	7	7	0.55	1
OCD 2032	✓	✓	✓	✓	2	47	31	16	15	8	27	14	13	0.13	0
OCD 569	✓	✓	✓	✓	1	49	32	16	16	9	22	11	11	0.31	0
OCD 884	✓	✗	✓	✓	2	41	32	17	15	16	24	12	12	0.25	0
OCD 1001	✗	✗	✓	✓	1	26	34	17	17	13	30	15	15	0.12	0
OCD 992	✓	✓	✓	✗	1	54	36	18	18	24	32	16	16	0.11	0
OCD 994	✓	✗	✗	✗	1	21	36	18	18	22	16	8	8	0.56	1
OCD 2022	✗	✗	✓	✓	2	59	37	19	18	8	34	17	17	0.08	0
OCD 1021	✓	✓	✓	✓	1	51	38	19	19	16	30	15	15	0.21	0
**Total ✓**	32	31	32	32											
**Total ✗**	11	12	11	11											
**Total**	43	43	43	43											

Gender (F = female, M = male); Y-BOCS T0 = Yale–Brown Obsessive Compulsive Scale at time 0 (before treatment); Y-BOCS Obs T0 = Yale–Brown Obsessive Compulsive Scale, Obsessions Subscale at time 0 (before treatment); Y-BOCS Comp T0 = Yale–Brown Obsessive Comp Compulsions Subscale at time 0 (before treatment); HRDS T0 = Hamilton Depression Rating Scale at time 0 (before treatment); Y-BOCS T3 = Yale–Brown Obsessive Compulsive Scale at time 3 (after treatment); Y-BOCS Obs T3 = Yale–Brown Obsessive Compulsive Scale Obsessio time 3 (after treatment); Y-BOCS Comp T3 = Yale–Brown Obsessive Compulsive Scale Compulsions Subscale at time 3 (after treatment); Response = YBOCS T0 - YBOCS T3/Y-BOCS T0 > 0,35 (0=No, 1= Yes); HRDS T3 = Hamilton Depression Rating Scale at time 3 (after treatmer family history of OCD ((0= No, 1 = Yes); Comorbidity (0= No, 1= Depressive Disorders; 2= Anxiety Disorders other than OCD, 3: Attention Deficit and Hiperactivity Disorder); Tics: Tics Disorders (0 = No, 1 = Yes); ResLevel = Level of Treatment Resistance previous to current treatm patient, 1: resistance to one SSRI, 2= resistance to 2 or more SSRIs, 3= resistance to clomipramine, 4= resistance to antipsychotic potentiation of SSRI or clomipramine); ObsAgr = aggressive obssesions (0 = no symptoms, 1 = present symptom, 2 = main symptom); ObsCont = Co obsessions (0 = no symptoms, 1 = present symptom, 2 = main symptom); ObsSex = Sexual obsessions (0 = no symptoms, 1 = present symptom, 2 = main symptom); ObsAccum = Hoarding obsessions (0 = no symptoms, 1 = present symptom, 2 = main symptom); ObsRel = Religious = no symptoms, 1 = present symptom, 2 = main symptom); ObsSim = Symmetry obsessions (0 = no symptoms, 1 = present symptom, 2 = main symptom); ObsMis = Miscellaneous obsessions (0 = no symptoms, 1 = present symptom, 2 = main symptom); ObsSom = Somatic obses symptoms, 1 = present symptom, 2 = main symptom); CompClean = cleaning/washing compulsions (0 = no symptoms, 1 = present symptom, 2 = main symptom); com_cont (Counting Compulsions: 0 = no symptoms, 1 = present symptom, 2 = main symptom); com_comp (Checking C = no symptoms, 1 = present symptom, 2 = main symptom); RitRep = Repeating Compulsions (0 = no symptoms, 1 = present symptom, 2 = main symptom); CompOrd = Ordering compulsions (0 = no symptoms, 1 = present symptom, 2 = main symptom); ComAccum = Hoarding comp symptoms, 1 = present symptom, 2 = main symptom); CompMis = miscellaneous compulsions (0 = no symptoms, 1 = present symptom, 2 = main symptom).

HRDS T3	FamOCD	Comorbidities	Tics	Age of onset	ResLevel	ObsAgr	ObsCont	ObsSex	ObsAccum	ObsRel	ObsSim	ObsMis	ObsSom	CompClean	com_cont	com_comp	RitRep	CompOrd	CompAccum
7	0	0	0	15	0	2	2	0	0	0	0	0	0	2	0	2	0	0	0
7	1	2	0	15	1	2	0	1	0	0	1	0	1	0	0	2	1	1	0
5	0	0	0	25	0	1	2	0	1	0	1	0	0	2	1	1	1	1	1
13	0	3	0	16	0	1	1	2	1	0	0	0	1	1	1	1	1	0	1
7	1	0	0	30	1	2	0	0	1	0	0	0	0	0	0	2	1	1	1
10	1	2	0	6	1	0	2	1	0	0	0	0	0	2	0	1	0	1	0
6	1	0	1	14	1	2	0	0	0	0	0	1	0	0	1	2	1	0	0
14	0	1	0	12	1	0	2	1	0	0	1	0	0	2	0	0	1	1	0
4	0	0	0	69	0	2	0	0	0	0	1	0	0	0	0	0	0	0	0
11	0	0	0	37	2	0	2	0	0	0	0	0	0	2	0	0	0	0	0
9	0	0	0	16	2	0	0	0	0	0	2	0	0	0	0	0	2	2	0
8	1	1	0	16	3	2	0	0	0	0	0	0	0	0	0	2	0	0	0
11	0	0	1	14	2	2	1	0	1	1	0	0	0	1	1	2	1	0	1
12	0	0	0	25	4	2	0	1	0	0	0	0	0	1	0	2	2	0	0
15	0	2	0	27	4	1	2	0	0	0	2	0	0	2	0	1	1	2	0
8	0	0	0	33	1	1	2	0	0	0	0	0	0	2	0	2	0	0	0
17	1	0	0	15	4	1	2	0	0	0	0	0	0	2	0	1	1	1	0
13	0	0	1	14	2	1	1	0	0	0	1	0	0	1	0	1	1	1	0
7	0	0	0	15	0	0	2	1	0	0	1	0	0	2	1	1	0	0	0
9	0	0	0	21	2	0	0	0	0	0	2	0	0	0	0	0	1	2	0
15	1	1	0	38	4	1	2	0	0	0	1	0	0	2	0	1	0	2	0
7	0	0	0	15	1	2	1	0	0	0	1	0	0	1	0	2	1	1	0
9	1	2	0	6	1	1	2	0	0	0	0	1	1	2	1	1	1	1	0
10	0	2	0	10	2	1	0	0	0	0	2	0	0	0	0	1	1	2	0
13	0	1	0	17	0	2	0	0	0	0	0	0	0	0	0	2	0	0	0
14	0	2	0	20	2	2	1	0	0	0	0	0	0	1	1	2	0	0	0
15	0	0	0	10	2	2	0	0	0	0	0	0	0	0	1	0	1	0	0
10	0	0	0	25	1	2	0	0	0	0	0	0	0	0	2	2	1	0	0
7	0	0	0	19	0	1	1	0	0	0	0	2	1	1	0	2	0	0	0
6	0	2	0	10	4	2	0	0	0	0	1	1	1	0	0	2	0	0	0
7	0	0	0	37	1	0	1	0	0	0	2	0	0	1	0	0	0	2	0
8	1	1	1	23	2	2	0	0	0	0	1	0	0	0	1	2	1	1	0
11	0	1	1	23	4	1	2	0	0	0	0	0	0	2	0	1	0	0	0
11	0	0	1	16	3	1	2	0	0	0	0	0	0	2	0	0	1	0	0
21	0	1	0	29	2	2	0	0	0	0	0	0	0	0	0	2	0	0	0
14	0	1	0	13	2	1	0	0	0	1	0	0	0	0	0	2	1	0	0
8	0	0	0	27	1	2	0	0	1	0	0	1	0	0	0	2	1	0	1
14	0	0	0	6	1	1	0	0	0	0	2	0	0	0	1	0	2	1	0

ɔulsive Scale,
ns Subscale at
ıt); FamOCD =
ent (0= naive
ntamination
; obsessions (0
sions (0 = no
Compulsions, 0
ulsions (0 = no

CompMis
0
0
0
0
0
0
0
0
2
0
0
0
0
0
0
0
0
0
1
0
0
0
0
0
0
0
0
0
2
1
0
0
0
0
0
1
1
0

Supplementary Table S2. Clinical data from control samples

Name	Stool sample	Pharyngeal swab sample	Gender	Age	Other medical conditions	Current medication
Control 91	✓	✓	2	27	Gastroesophageal reflux disease; Polycystic ovary syndrome; Liver hemangioma	
Control 92	✓	✓	1	28		
Control 93	✓	✓	2	26		
Control 97	✓	✓	1	26		
Control 98	✗	✗	2	26		
Control 99	✓	✓	2	28		
Control 100	✓	✓	2	47	Osteoarthritis; Vitiligo	
Control 101	✓	✓	1	52		
Control 102	✓	✗	1	50	Polycystic kidney disease; Arterial hypertension; Meniscectomy	Arterial hhypertension treatment
Control 103	✓	✓	2	24		
Control 104	✓	✓	2	24		
Control 105	✓	✓	2	24		
Control 106	✓	✓	2	23		
Control 107	✓	✓	2	25		
Control 108	✓	✓	2	26		
Control 109	✓	✓	2	31		
Control 110	✓	✓	2	48		
Control 111	✓	✓	2	37		Contraceptives
Control 112	✓	✓	1	47		
Control 113	✓	✓	2	42		
Control 114	✓	✓	1	29		
Control 115	✓	✓	1	59		
Control 116	✓	✓	1	34		
Control 117	✓	✓	2	32		
Control 118	✓	✓	2	35		
Control 119	✓	✓	2	47		
Control 120	✓	✓	1	44		
Control 121	✓	✓	1	37	Bronchial asthma	Budesonide; Salbutamol
Control 122	✓	✓	1	46		
Control 123	✓	✓	1	33		
Control 124	✓	✓	2	41	Post-pregnancy hyperthyroidism (now asymptomatic)	
Control 125	✓	✓	1	33		
Control 126	✓	✓	1	42		
Control 127	✓	✓	1	41		
**Total ✓**	33	32				
**Total ✗**	1	2				
**Total**	34	34				

Gender: F = female, M = male;

Supplementary tables:

Table S3. Diet questionnaire

	Dietary	Did you eat or drink the following products in the last 7 days?	If yes, how frequent? (Please choose only one response per category, from this column)
**Q1**	Tea or coffee no sugar and no sugar replacement	○ No, I did not consume these products in the last 7 days	○ Once a week<br>○ 3 to 4 times at week<br>○ Daily
**Q2**	Soft drinks, tea or coffee with sugar (corn syrup, maple syrup, cane sugar, etc.)	○ No, I did not consume these products in the last 7 days	○ Once a week<br>○ 3 to 4 times at week<br>○ Daily
**Q3**	Diet soft drinks, tea or coffee with sugar substitute (Stevia, Equal, Splenda, etc.)	○ No, I did not consume these products in the last 7 days	○ Once a week<br>○ 3 to 4 times at week<br>○ Daily
**Q4**	Fruit juice (orange, apple, cranberry, prune, etc.)	○ No, I did not consume these products in the last 7 days	○ Once a week<br>○ 3 to 4 times at week<br>○ Daily
**Q5**	Water	○ No, I did not consume these products in the last 7 days	○ Once a week<br>○ 3 to 4 times at week<br>○ Daily
**Q6**	Alcohol (beer, brandy, spirits, hard liquor, wine, aperitif, etc.)	○ No, I did not consume these products in the last 7 days	○ Once a week<br>○ 3 to 4 times at week<br>○ Daily
**Q7**	Yogurt or other foods containing active bacterial cultures (Actimel, kefir, sauerkraut, etc.)	○ No, I did not consume these products in the last 7 days	○ Once a week<br>○ 3 to 4 times at week<br>○ Daily
**Q8**	Dairy (milk, cream, ice cream, cheese, cream cheese)	○ No, I did not consume these products in the last 7 days	○ Once a week<br>○ 3 to 4 times at week<br>○ Daily
**Q9**	Probiotic (other than yogurt)	○ No, I did not consume these products in the last 7 days	○ Once a week<br>○ 3 to 4 times at week<br>○ Daily
**Q10**	Fruits (frozen or fresh, no juice) (apples, raisins, bananas, oranges, strawberries, blueberries, etc.)	○ No, I did not consume these products in the last 7 days	○ Once a week<br>○ 3 to 4 times at week<br>○ Daily
**Q11**	Vegetables (salad, tomatoes, onions, greens, carrots, peppers, green beans, etc.)	○ No, I did not consume these products in the last 7 days	○ Once a week<br>○ 3 to 4 times at week<br>○ Daily
**Q12**	Beans and beans sprouts (tofu, soy, soy burgers, lentils, Mexican beans, lima beans, etc.)	○ No, I did not consume these products in the last 7 days	○ Once a week<br>○ 3 to 4 times at week<br>○ Daily
**Q13**	Whole grains (wheat, oats, brown rice, rye, wheat bread, wheat pasta, etc.)	○ No, I did not consume these products in the last 7 days	○ Once a week<br>○ 3 to 4 times at week<br>○ Daily
**Q14**	Starch (white rice, bread, pizza, potatoes, yam, cereals, pancakes, etc.)	○ No, I did not consume these products in the last 7 days	○ Once a week<br>○ 3 to 4 times at week<br>○ Daily

Q= Question

Table S3 (continued). Diet questionnaire

	Dietary	Did you eat or drink the following products in the last 7 days?	If yes, how frequent? (Please choose only one response per category, from this column)
**Q15**	Eggs	○ No, I did not consume these products in the last 7 days	○ Once a week<br>○ 3 to 4 times at week<br>○ Daily
**Q16**	Processed meat (other red meat and other white meat such as lunch meat, ham, salami, bologna, sausage, kielbasa) hotdog, bacon, etc.)	○ No, I did not consume these products in the last 7 days	○ Once a week<br>○ 3 to 4 times at week<br>○ Daily
**Q17**	Red meat (beef, hamburger, pork, lamb)	○ No, I did not consume these products in the last 7 days	○ Once a week<br>○ 3 to 4 times at week<br>○ Daily
**Q18**	White meat (chicken, turkey, etc.)	○ No, I did not consume these products in the last 7 days	○ Once a week<br>○ 3 to 4 times at week<br>○ Daily
**Q19**	Shellfish (shrimp, lobster scallops, etc.)	○ No, I did not consume these products in the last 7 days	○ Once a week<br>○ 3 to 4 times at week<br>○ Daily
**Q20**	Fish (fish nuggets, breaded fish, fish cakes, salmon, tuna, etc.)	○ No, I did not consume these products in the last 7 days	○ Once a week<br>○ 3 to 4 times at week<br>○ Daily
**Q21**	Sweets (pies, jam, chocolate, cake, cookies, etc.)	○ No, I did not consume these products in the last 7 days	○ Once a week<br>○ 3 to 4 times at week<br>○ Daily

Q= Question

Table S4: summary responses to diet questionnaire

	Q	1	2	3	4	5	6	7	8	9	10	11	12	13	14	15	16	17	18	19	20	21
Answer OCD	1	29	17	30	18	1	24	14	5	35	3	1	3	18	0	4	6	7	2	26	10	2
	2	0	6	2	10	0	10	3	2	0	4	7	26	8	10	20	14	22	16	10	21	7
	3	1	2	1	3	0	4	11	4	1	12	18	7	7	12	13	14	8	19	2	7	15
	4	8	13	4	6	36	0	10	27	1	19	12	2	5	16	0	4	1	1	0	0	14
Answer Control	1	19	10	22	10	0	7	7	0	30	1	0	5	4	0	4	3	6	3	15	2	2
	2	1	4	3	12	0	15	9	4	1	4	1	20	8	4	15	11	18	12	15	17	6
	3	2	8	3	5	0	8	11	9	0	7	10	7	12	17	12	15	8	15	1	12	15
	4	10	10	4	4	32	2	5	19	0	20	21	0	8	11	1	3	0	2	0	1	9

Supplementary Table 5. Individual abundace for top taxa in stool samples

LEFSE HIGHLIGTED	TOC1000.T0	TOC1000.T3	TOC1002.T0	TOC1002.T3	TOC1011.T0	TOC1012.T0	TOC1012.T3	TOC1014.T0	TOC1014.T3	TOC1021.T0
Bacteria | Bacteroidetes | Bacteroidia | Bacteroidales | Rikenellaceae | Alistipes | Alistipes finegoldii	0.0842124	0	0.2364915	0.4592972	0	0	0.0866185	0.6175278	0.0891795	0.2174893
Bacteria | Bacteroidetes | Bacteroidia | Bacteroidales | Rikenellaceae | Alistipes | Alistipes ihumii	0.0443223	0	0	0	0	0	0	0.1122778	0.0571664	0
Bacteria | Bacteroidetes | Bacteroidia | Bacteroidales | Rikenellaceae | Alistipes | Alistipes indistinctus	0	0	0	0	0	0	0	0	0	0
Bacteria | Bacteroidetes | Bacteroidia | Bacteroidales | Rikenellaceae | Alistipes | Alistipes inops	0	1.0848558	0	0	0	0	0.0555247	0	0	0
Bacteria | Bacteroidetes | Bacteroidia | Bacteroidales | Rikenellaceae | Alistipes | Alistipes obesi	0	0.5024867	0.3262985	0.0478435	0	0.0711335	0.0866185	0.2391101	0.0777463	0.4016694
Bacteria | Bacteroidetes | Bacteroidia | Bacteroidales | Rikenellaceae | Alistipes | Alistipes putredinis	1.5446326	0	0.4550217	0.5478076	0	0.2432308	0.2509717	3.0917975	1.7950242	0
Bacteria | Bacteroidetes | Bacteroidia | Bacteroidales | Rikenellaceae | Alistipes | Alistipes senegalensis	0	0	0	0	0	0	0	0	0	0
Bacteria | Bacteroidetes | Bacteroidia | Bacteroidales | Rikenellaceae | Alistipes | Alistipes shahii	0	0	0	0	0.1	0.1766866	0.2642976	0.3680216	0	0.885632
Bacteria | Bacteroidetes | Bacteroidia | Bacteroidales | Rikenellaceae | Alistipes | Alistipes unclassified.S187	2.377892	1.5615739	0.047897	0.083726	0	0.4199174	0.3331483	0.63832	0.3086984	0
Bacteria | Bacteroidetes | Bacteroidia | Bacteroidales | Rikenellaceae | unclassified.G52 | unclassified.S192	0	0	0	0	0	0.0045893	0	0	0	0
Bacteria | Bacteroidetes | Bacteroidia | Bacteroidales | Barnesiellaceae | Coprobacter | Coprobacter secundus	0.1041574	0	0.0628648	0	0.0447368	0	0	0	0	0
Bacteria | Bacteroidetes | Bacteroidia | Bacteroidales | Barnesiellaceae | Coprobacter | Coprobacter unclassified.S178	0.0664835	0.0334991	0	0.0215296	0.0210526	0	0	0.2453477	0.1371993	0.0529028
Bacteria | Bacteroidetes | Bacteroidia | Bacteroidales | Barnesiellaceae | Barnesiella | Barnesiella intestinihominis	0	0	0	0	0	0.043598	0	0	0	0
Bacteria | Bacteroidetes | Bacteroidia | Bacteroidales | Barnesiellaceae | Barnesiella | Barnesiella unclassified.S177	0	0.5462932	1.4668463	0.6315336	0	0	0	0.8961431	0.715723	0.8973882
Bacteria | Bacteroidetes | Bacteroidia | Bacteroidales | Barnesiellaceae | unclassified.G51 | unclassified.S179	0.037674	0.1571881	0	0	0	0.0367141	0.1288173	0	0	0
Bacteria | Bacteroidetes | Bacteroidia | Bacteroidales | Prevotellaceae | Prevotella | Prevotella unclassified.S33	0	0	0	0	45.828947	21.778339	3.3914492	0	0	0
Bacteria | Bacteroidetes | Bacteroidia | Bacteroidales | Prevotellaceae | Prevotellaceae_NK3B31_group | Prevotellaceae_NK3B31_group unclassified.S184	0	0	0	0	0.5657895	0	0	0	0	0
Bacteria | Bacteroidetes | Bacteroidia | Bacteroidales | Prevotellaceae | unclassified.G7 | unclassified.S35	0	0	0	0	0.1184211	0.0734282	0	0	0	0
Bacteria | Bacteroidetes | Bacteroidia | Bacteroidales | Prevotellaceae | Paraprevotella | Paraprevotella unclassified.S182	0	0	0	0	0	0.5300597	0.9905608	0	0	1.5224249
Bacteria | Firmicutes | Clostridia | Clostridiales | Lachnospiraceae | Anaerostipes | Anaerostipes hadrus	0.1285347	0.0901899	0.8112558	0.3157668	0	0.1262047	0	0.1268323	0	0.7523953
Bacteria | Firmicutes | Clostridia | Clostridiales | Lachnospiraceae | Anaerostipes | Anaerostipes unclassified.S220	0	0	0.3023499	0	0	0	0.0777346	0	0.3544315	0.1273586
Bacteria | Firmicutes | Clostridia | Clostridiales | Lachnospiraceae | Agathobacter | Agathobacter unclassified.S70	8.1796827	0.7498647	1.4997755	3.0141377	4.1578947	2.8407526	4.8661855	5.5993347	5.4376658	5.9760566
Bacteria | Firmicutes | Clostridia | Clostridiales | Lachnospiraceae | Coprococcus | Coprococcus catus	0	0	0.0838198	0.1841973	0	0	0	0.0499012	0.0960395	0
Bacteria | Firmicutes | Clostridia | Clostridiales | Lachnospiraceae | Coprococcus | Coprococcus comes	0.4831132	0.0953436	0.8352043	0.5621606	0.3105263	0.3900872	0.2287618	0.3222788	0.4115979	0.4898407
Bacteria | Firmicutes | Clostridia | Clostridiales | Lachnospiraceae | Coprococcus | Coprococcus eutactus	0	0	0	0	0	0.0596604	0	0	0	0
Bacteria | Firmicutes | Clostridia | Clostridiales | Lachnospiraceae | Coprococcus | Coprococcus unclassified.S223	0	0	0.1496782	0.0382748	0.6815789	0	0	0	0	0
Bacteria | Firmicutes | Clostridia | Clostridiales | Lachnospiraceae | Hungatella | Hungatella hathewayi	0.1817215	0	0.0628648	0	0	0	0	0	0	0.2037737
Bacteria | Firmicutes | Clostridia | Clostridiales | Lachnospiraceae | Howardella | Howardella unclassified.S74	0	0	0	0.05502	0.0289474	0.0413034	0.0444198	0	0	0
Bacteria | Firmicutes | Clostridia | Clostridiales | Lachnospiraceae | Lachnospiraceae_FCS020_group | Lachnospiraceae_FCS020_group unclassified.S235	0	0	0.1706331	0.1554912	0	0	0	0	0	0
Bacteria | Firmicutes | Clostridia | Clostridiales | Lachnospiraceae | Lachnospira | Lachnospira pectinoschiza	1.2232958	0.9972428	1.0118246	0.7272205	2.3763158	0.4887563	1.1549139	0.1497037	0	2.3336011
Bacteria | Firmicutes | Clostridia | Clostridiales | Lachnospiraceae | Lachnospira | Lachnospira unclassified.S233	0.0066483	1.8965651	1.7033378	2.1840538	1.5184211	3.3249197	4.2620766	0.428319	4.2303119	0
Bacteria | Firmicutes | Clostridia | Clostridiales | Ruminococcaceae | Oscillibacter | Oscillibacter unclassified.S270	0.3523624	0.2035715	0.0838198	0.0741574	0.0473684	0.0573658	0.0866185	0.5801019	0.1417726	1.5126281
Bacteria | Firmicutes | Clostridia | Clostridiales | Ruminococcaceae | Flavonifractor | Flavonifractor plautii	0	0	0.1257297	0.3157668	0.3394737	0	0	0.0894064	0.0480198	1.1795364
Bacteria | Firmicutes | Clostridia | Clostridiales | Ruminococcaceae | Flavonifractor | Flavonifractor unclassified.S265	0.8354756	0	0	0	0.0763158	0	0	0	0.0342998	0
Bacteria | Firmicutes | Clostridia | Clostridiales | Ruminococcaceae | UBA1819 | UBA1819 unclassified.S287	0.7512632	0.1855335	0.068852	0.0454513	0	0.0344195	0.0355358	0.0499012	0.0754596	0.2468797
Bacteria | Firmicutes | Clostridia | Clostridiales | Ruminococcaceae | Ruminococcaceae_UCG-002 | Ruminococcaceae_UCG-002 bacterium	0	0	4.056279	2.408918	0	0.826067	1.7701277	0	0.3452849	0
Bacteria | Firmicutes | Clostridia | Clostridiales | Family_XIII | Family_XIII_AD3011_group | Family_XIII_AD3011_group unclassified.S217	0	0	0	0.0239217	0	0.0688389	0.2154359	0	0	0
Bacteria | Firmicutes | Clostridia | Clostridiales | Family_XIII | Family_XIII_UCG-001 | Family_XIII_UCG-001 unclassified.S64	0	0.0231917	0	0	0	0	0.0111049	0	0	0
Bacteria | Firmicutes | Clostridia | Clostridiales | Family_XIII | unclassified.G20 | unclassified.S66	0	0.0309223	0	0	0	0	0	0	0	0
Bacteria | Firmicutes | Clostridia | Clostridiales | Clostridiaceae_1 | Clostridium | Clostridium unclassified.S211	0	0	0	0.2368251	0.0947368	0	0.1265963	0	0.0091466	0
Bacteria | Firmicutes | Clostridia | Clostridiales | Peptostreptococcaceae | Romboutsia | Romboutsia unclassified.S253	0	0	0.0179614	0.6961223	0.2815789	0.0711335	0.0488617	0	0	0
Bacteria | Firmicutes | Negativicutes | Selenomonadales | Acidaminococcaceae | Acidaminococcus | Acidaminococcus intestini	2.2338445	0	0	0	0	0	0.0310938	0	0	0
Bacteria | Firmicutes | Negativicutes | Selenomonadales | Acidaminococcaceae | Phascolarctobacterium | Phascolarctobacterium faecium	0	0	0	0	0	0	0	1.0354507	0.9924083	0.8895507
Bacteria | Firmicutes | Negativicutes | Selenomonadales | Acidaminococcaceae | Phascolarctobacterium | Phascolarctobacterium unclassified.S308	0	0.5746386	0	0	0	1.1197797	1.0638534	0	0.0205799	0.2233674
Bacteria | Firmicutes | Negativicutes | Selenomonadales | Veillonellaceae | Allisonella | Allisonella unclassified.S311	0	0	0	0	0	0.0527765	0	0	0	0
Bacteria | Firmicutes | Negativicutes | Selenomonadales | Veillonellaceae | Veillonella | Veillonella unclassified.S104	0.1551281	0	0	0	0	0.018357	0	0	0.0182932	0
Bacteria | Firmicutes | Negativicutes | Selenomonadales | Veillonellaceae | Dialister | Dialister invisus	0.7601276	0	1.1405478	0.5860823	2.1684211	0	0	0	0	0
Bacteria | Firmicutes | Negativicutes | Selenomonadales | Veillonellaceae | Dialister | Dialister unclassified.S314	0	0	0.8232301	1.4544411	0	0	0	0	0	0
Bacteria | Firmicutes | Negativicutes | Selenomonadales | Veillonellaceae | unclassified.G24 | unclassified.S103	0.0044322	0	0	0	0	0	0	0	0	0
Bacteria | Firmicutes | Erysipelotrichia | Erysipelotrichales | Erysipelotrichaceae | Catenibacterium | Catenibacterium unclassified.S293	0	0	0	0	0	0.4153281	0.3553581	0	0	0
Bacteria | Tenericutes | Mollicutes | Mollicutes_RF39 | unclassified.F36 | unclassified.G77 | unclassified.S358	0	0	0	0	0	0.0137678	0	0	0	0
Bacteria | Lentisphaerae | Lentisphaeria | Victivallales | vadinBE97 | unclassified.G66 | unclassified.S318	0	0	0	0	0	0	0	0	0	0

TOC1021.T	TOC1025.T(	TOC1025.T	TOC1027.T(	TOC1027.T	TOC1030.T(	TOC1030.T	TOC1031.T(	TOC1031.T	TOC2017.T(	TOC2017.T	TOC2020.T(	TOC2020.T	TOC2024.T(	TOC2024.T	TOC2026.T(	TOC2026.T	TOC2027.T(	TOC2027.T	TOC2029.T(	TOC2029.T	TOC2030.T	TOC2031.T(	TOC2031.T
0.525478	0	0	0.3910221	0.8857787	0	0	0.1432045	0.1166609	0	0	0.2580224	1.6781451	0.8823993	0.2622616	0	0	0.8182748	0.7813188	0.089733	0.2096701	0.1961978	0.5736258	0.0765148
0	0	0	0	0	0.0171518	0.3027163	0	0	0.0798551	0.2820504	0	0	0	0	0	0	0	0	0	0	0	0	0
0	0	0	0	0	0	0	0.3377466	0.1425856	0.0097384	0.0196779	0.1067679	0	0	0	0	0	0	0.2467323	0	0	0.0653993	0	0
0	0.2815485	0.3755011	0.1251271	0.5300723	0	0.0272717	0	0	0.9699472	0.2722115	0	0	0	0	0	0	0	0	0.1171515	0	0.2164941	0	0
0.0364383	0	0.0989496	0	0.3301328	0	0.0381804	0.0918671	0.0432077	0.1694486	0.2361353	0	0.3508511	0.0490222	0.0374659	0.0360642	0.1294045	0.1153977	0.925246	0.1744809	0.3915526	0	0	0
0	0	0	2.1897239	0	0.5219053	2.986255	0	0	0	0	2.1828104	0.1522211	2.3040426	2.7588556	2.688786	4.9200658	2.7800362	3.5981789	2.520003	3.1501036	2.6655842	2.4063918	1.7383195
0	0	0	0	0.1325181	0	0	0	0	0	0	0	0	0	0	0	0	0	0	0	0	0	0	0
0.2953417	0	0	0	0	0	0	0	0.0604908	0	0	0	0	0	0	0	0	0	0	0	0.1515687	0.8885281	0	0
0	0.2681414	0.2714771	13.881286	1.185688	0.3479369	6.2452274	1.5482302	0.2030764	1.3068968	1.4988029	3.4373332	2.9794501	0.6880613	0.1907357	0.2945243	0.3612541	0.2203048	0.7490087	1.1067075	0.6012227	4.0412241	5.4447176	0.6695041
0	0	0	0	0	0	0.0190902	0	0	0	0.0147585	0	0	0	0	0	0	0	0.0058746	0	0	0	0	0
0	0.0234624	0.0380575	0	0	0	0.0599978	0	0.0475285	0	0	0	0	0.0962936	0.0698229	0	0	0	0	0	0	0.0383375	0	0
0.028767	0.043573	0.0076115	0.0130341	0.0883454	0	0.0272717	0	0	0.0506398	0.0426355	0.9668426	0	0.008754	0	0.0440785	0.07279	0	0.0675576	0.0373888	0.0252615	0	0.2237771	0.2964947
0	0	0	0	0	0	0	0	0	0	0	0	0.9690174	0	0	0	0	0	0	0	0	0	0	0
0.1917803	0.7843137	0.3729639	1.136571	0	0	0.8590597	0.0135099	0.0129623	0.5979393	1.6070316	0	0.0074254	0	0	0.2905171	0.4717871	0.6241968	1.4304597	0.1969142	0.2399838	0.719392	0	0
0	0	0	0.1146998	0.306884	0	0	0	0.133944	0	0	0	0.2487516	0	0	0	0	0	0	0.0423739	0.0277876	0.1262883	0	0
0	0	0	0	0	48.162305	0.6272499	0.9619022	11.933979	0	0	0	0	0	0	0	0	0	0	0	0	0	0	0
0	0	0	0	0	0	0	0	0	0	0	0	0	0	0	0	0	0	0.5757086	0	0	0	0	0
0	0	0	0	0	0.0147016	0	0	0	0	0	0	0	0	0	0	0	0	0	0	0	0	0	0
1.8468443	0	0	0	0	0.8845438	0.4663467	0	0.3715866	0	0	1.2337624	0	0	0	0	0	0	0	0	0	0	0	0
0.3010951	0.2145132	0.4566905	0.1798702	0	0	0	0	0.0432077	0.8550338	0.1672625	0	0.8260781	0.2241014	0.0425749	1.0759151	0.5957997	0	0	0.0797627	0.4041833	0.0631441	0	0.3490986
0	0	0	0	0.0627717	0	0	0	0	0.3856417	0	0	0.1615029	0.1873348	0.0374659	0	0	0.5402712	0.5727713	0	0	0	0.0929778	0
5.8723127	1.8434724	12.690922	3.9024009	0.7021133	1.631873	5.3152613	0.0621454	0.3370204	0.1830824	0.4739103	0	0.679426	0.0437698	0	1.4085071	6.4405683	3.0186997	1.7036276	0.4411875	1.960289	0.780281	0	3.0797188
0	0.0368694	0.0964124	0.0573499	0	0.0686073	0	0.2134558	0.0864155	0.1460764	0.0967499	0	0	0	0	0.1222175	0	0.0419628	0	0.0648072	0	0.0563787	0	0
0	0.4860064	0.8372659	0.041709	0.2789854	0.4116436	0	0	0.3413412	0	0	0.1097337	0	0.1680761	0.2826975	1.3984893	1.4719758	0.3488159	0.0793068	0.1645106	0.7376345	0.214239	0	0
0	0	0	0	0	0	0	0	0	0.0934889	0	0	0	0	0	0	0	0	0	0	0.156621	0	0	0
0	0	0	0.6960194	0.0139493	0	0	0.6917049	0	0.1304949	0	0	0	0	0	0	0	0	0	0.7103866	0	0	0.2017146	0
0.0747943	0	0	0	0	0	0	0.9294785	0	0	0.429635	0.1423572	0.0278453	0.0245111	0	0	0	0	0	0	0	0	0.179652	0
0	0	0	0	0	0	0	0	0	0	0	0	0	0.0332651	0	0	0	0	0	0	0	0.1150125	0	0
0	0	0	0	0	0.1004606	0	0	0	0	0	0	0	0	0	0.2344173	0.1994986	0.1075297	0.0469966	0.0423739	0	0	0	0
1.2408185	0.5865594	2.3240473	1.0740075	0	0.2082721	0.329988	0.294515	0.440719	0.1090704	0.4853891	0	0	2.2812823	2.1355586	0.8254693	5.2732321	0.8104068	0.5345866	0.3016027	1.0432981	0.8389148	0.5011346	4.7128306
0.9761617	0.3284733	1.3117166	2.9457001	0.9903983	0.7938842	4.636195	0.591732	0.3586243	0.7031143	0.1771014	0	0	0.0035016	0	0.6251127	2.1378697	7.8024601	1.2424732	0.4287245	1.0306674	1.3170061	0	0
0.1879447	0.1206637	0.3069975	0.3675608	0.7300119	0	0.3108978	0.0648473	0.267888	0.1636056	0.3755206	1.8209858	0.8297908	0.6425407	0.0715259	0.1422532	0.2453293	0.4694589	0.1498017	0.1196441	0.2879806	0.658503	1.3300555	0.2821482
0.5695875	0.5899112	0.1268585	0.0834181	0.0418478	0	0	0	0	0.0798551	0.0852711	0.2461593	0.0389835	0.2906315	0.4870572	0.2624672	0.6470223	0	0	0.1470625	0.3359774	0.0721647	0.5846571	1.279231
0.0863011	0	0	0.028675	0.058122	0.0465549	0.1718119	0	0.1296232	0.0350583	0.0311567	0.2224331	0.0798233	0.1050475	0	0.0440785	0	0.1232658	0	0	0	0.0902059	0.0567322	0.1267276
0.0728765	0.1072566	0.1217841	0.1485884	0.2162137	0	0.1118141	1.5401243	0.1857933	0.0447968	2.0563445	0.6228127	0.224619	0.2661204	0.027248	0	0.0862696	0.0813029	0.0939932	0.0997034	0.2324054	0.2390456	0.5137418	0.0717326
0.0421917	0.8446456	1.436038	0.6517036	0.325483	0.3626384	1.2490455	0	5.91514	0	0	0	0	0.6705534	0.6641689	2.2459979	0	2.2581237	0.6638273	0	0	0.5637868	0	0
0.0172602	0.0335177	0.0888009	0.0860249	0.1952898	0	0	0.1459065	0.2549257	0	0.0409957	0	0.4102545	0	0	0.0641141	0.0647022	0.089171	0.0939932	0.0697924	0	0.1195228	0.6744831	0
0.0594519	0	0.0888009	0.0521363	0.0302234	0.0171518	0	0	0	0	0	0	0.031558	0.0490222	0.0749319	0.046082	0.0242633	0	0.0234983	0	0	0.2007081	0.0220625	0.0239109
0	0	0.0202974	0	0	0	0	0	0	0	0.0114788	0	0.0167072	0	0	0	0	0	0.0528712	0	0	0	0.0063036	0
0	0	0	0	0	0.5905126	0.1090869	0	0	0	0	0	0	0	0.018733	0.0160285	0	0	0	0	0	0	0	0
0	0	0	0	0	0.4116436	0.0981782	0	0	0	0	0	0	0	0	0	0	0	0	0	0	0	0	0
0	0	0	0	0	0	0	0	0	0	0	0	0	0	0	0	0	0.2150594	1.016302	0.3364989	0.0126307	0.2818934	0	0
0.6980803	1.1731188	0.0989496	0.7377284	0	0	0	0	0	0	0	0	1.1787856	0.084038	0	0.2825029	0.439436	0	0	0	1.0331936	0	0	0
0.2167117	0	0	0.0495295	0.5672704	0.5439577	0.329988	0	0	0	0	0	0.6961332	0.6267836	0.2980245	0	0	0.4143828	0.1174916	0	0.035366	0.6923302	0	0
0	0	0	0	0	0	0	0	0	0.0272676	0.0491948	0	0	0.0665301	0.1447548	0	0	0	0	0	0	0	0	0
0	0	0	0	0	0	0	0	0	0	0.037716	0	0	0	0	0.0220392	0.0970534	0	0	0	0	0	0	0
0	0	0	0	0	0	0	0	0	0.1090704	0.8149946	0.0355893	0	0	0	0	0	0	0	0.8250455	0	0	0.1623172	2.1711061
0	0	0.8854722	0	0	0	0	0	2.2640857	0	0.0295169	0	0	0	0	0	0	0	0	0	0	0	0.3104513	2.1424131
0	0	0	0	0	0	0	0	0.1901141	0	0	0	0.0037127	0	0	0	0	0	0	0	0	0	0	0
0	0	0	0	0	0	0	0.8889489	0.7302109	0	0	0	0	0	0	0	0	0	0	0	0	0	0	0
0	0	0	0	0	0	0	0	0	0	0	0	0	0	0	0	0	0	0	0.0099703	0	0	0	0
0	0	0	0	0.0255737	0	0	0	0	0	0	0	0	0	0	0	0	0	0	0	0	0	0	0

TOC2032.T(	TOC2032.T3	TOC569.T0	TOC569.T3	TOC644.T0	TOC644.T3	TOC834.T0	TOC834.T3	TOC875.T0	TOC875.T3	TOC884.T0	TOC885.T0	TOC885.T3	TOC893.T3	TOC896.T0	TOC897.T0	TOC897.T3	TOC898.T0	TOC898.T3	TOC899.T0	TOC899.T3	TOC953.T3	TOC972.T0	TOC972.T3
0	0	0	0	0	0	0	0.1682587	0	0	0.0557427	0.0449506	0.3796347	0.1689602	0.6460409	0.7164047	1.2520485	0.8402585	0	0.0302794	0	0.40006	0.0418523	0
0	0	0	0	0	0	0	0	0	0	0	0	0	0	0.1657053	0	0	0.1877501	0	0	0	0	0	0
0	0	0	0	0	0.029245	0	0	0.0734484	0.0593013	0.0766462	0	0	0	0	0	0.0218508	0	0.1559988	0.1557228	0	0.0483406	0	0
0	0	0.1091533	0.0989772	0.8221356	0.6861334	0	0	0	0	0.3251655	0.0374588	0.0590543	0	0.9732564	0	0	3.176362	0.4439964	1.07492	0.1522157	0	0.1046307	0.1086733
0.1858054	0.2501497	0.0592546	0.0681843	0.067865	0.1462251	0.0361942	0.7377495	1.7239972	0.6368441	0.5202648	0.0599341	0.0695997	0	0.1866807	0.198826	0	0.5109264	0.119999	0	0	0.0483406	0.1644196	0.0411196
0	0	1.6029939	2.0785219	0	0	0	0	1.3016689	0.6935671	0.6596214	0.3689691	0.7107605	0.9203886	0	0.6311936	1.0663171	0	0	0	2.355036	0.6767682	0.3647126	0.3553911
0	0	0	0	0	0	0.1432686	0.0906008	0.148937	0.0670362	0	0	0	0	0	0	0	0	0	0	0	0	0	0
0.7896731	0.6285812	0.0904413	0.1429671	0.2598259	0.3554396	0	0	0	0	0.262455	0.4382679	0.310035	0	0	0	0	0	0	0.1600484	0.1809357	0.0716774	0.3168814	0.0881135
1.3055277	0.5965107	0.3524092	0.8226108	1.9002191	2.7377846	0.2533593	0.6600916	0.9446281	1.0055434	0.870979	0.2247528	0.2235627	0.4668638	1.3613005	1.4233415	2.0233803	0.3601108	0.2855977	0.8694524	0.1550877	1.9836309	0.4902693	0.2055981
0	0.0128282	0	0.004399	0	0	0	0	0.0061207	0	0	0	0	0.0088926	0	0	0	0	0	0	0	0	0	0
0.0220033	0.0384846	0.0124747	0.017596	0	0	0	0	0	0	0.0278713	0	0	0	0	0	0	0	0	0	0	0	0	0
0.0660098	0	0.018712	0.017596	0.011634	0	0.0377023	0.0517719	0	0.0283615	0.0232261	0	0.2467626	0	0.1342423	0	0.2796897	0.0554017	0.0071999	0	0	0.0216699	0	0
0.344718	0.203113	0	0	0	0	0	0	0	0	1.6374405	0	0	0	0	0.2871931	0	0	0	0	0	0.4500675	0	0
1.6233528	0.7803814	0.2775612	0.2969317	0.065926	0.0494916	1.0828093	1.0690896	1.4281634	1.3252546	0	0.1629458	0.5462522	0.2645561	1.2857892	0	0	1.2988612	0	0.696427	0.0459519	0.2867097	0.4484171	0.2731518
0	0	0.0249493	0	0	0.0674885	0	0.0854236	0	0	0.2392289	0	0.078036	0.0222316	0	0.3219087	0	0.2092952	0	0	0.0516959	0.0133353	0.0478312	0.3583282
1.2688556	18.919439	0.3149852	2.8351479	0.4828108	0.0089985	0.1734305	0.0880122	0	0	0.0696783	0	0	0.2378782	0	0	0	0	0	4.4272861	0.9362704	8.0862129	0.0717467	0.2144095
0	0	0	0	0	0	0	0	0.0510058	0.056723	0	0.3146539	0.4766525	0.0444632	0	0	0	0	0.9383925	0	0	0	0	0
0	0	0.3555278	0.6048609	0	0	0	0	0	0	0	0	0	0	0	0	0	0	0	0.365516	0.4451593	0	0	0
0	0	0.8264463	0.3717145	0.1938999	0.2834518	0	0	0	0	0	0	0	0.0800338	0	0	0.2447285	0	0	0.5580067	0.5025992	0	0.0837045	0
0.5696404	0.4297443	0	0.2309469	0.100828	0.3306938	0.0723884	0.8076415	0	0.0876628	0.2136802	0.0824093	0.050618	0.0933728	0.2579969	0.2808811	0	0.1046476	0.5759954	0.0778614	0.0689279	0.0533413	0.1106095	0.4229448
3.7454465	3.9083212	0	0	0	0	0	0.5203075	0	0	0	0	0	0	0.5621395	0	0	0	0	0	0	0	0	0
1.4497714	1.637732	0.7048183	0.9127901	1.9719621	2.0966436	0	1.4314928	0	0.9565554	0.0557427	0.6143242	1.6493019	11.607123	0	0	4.3330056	2.70237	3.9719682	37.905528	3.7910336	0.2467037	1.0702221	2.2792023
0	0	0.0686106	0.0967777	0.056231	0.1259786	0	0.204499	0	0.0850844	0.0789688	0.0599341	0.0653815	0	0	0	0	0.0646353	0.251998	0.0778614	0.0430799	0.2850428	0.140504	0.2085353
0.6649879	0.3869837	0.4958678	1.2823051	0.3800438	0.1597228	0	0.1708472	0.2815522	0.1624339	0.2067123	0.247228	1.0714135	0.2912341	0	0	0.4785316	0	0.1775986	0.259538	0.8099026	0.1566902	0.1524618	0.4523159
0	0.4853331	0.8857009	0.6334543	0	0	0	0	0.3284776	0.1418074	0	0	0	0	0	0	0	0	0	0	4.8335679	0	0.3049236	0.3113343
0	0.0855213	3.0157493	3.556582	0	0.0247458	0	0	4.3497776	1.670749	0	0	0	0	0	0	0	0	0	0	1.2464459	0.0383391	1.3661774	1.2893941
0	0.0876593	0	0	0	0.0719878	0	0	0	0	0	0	0	0	0.085999	0	0.1420299	0.1261927	0.6959944	0	0.0603119	1.6685836	0	0
0	0	0	0	0	0	0	0	0.2019831	0.154699	0	0	0	0	0	0	0	0	0.1703986	0	0	0.040006	0.0418523	0.0704908
0	0	0	0.0505884	0	0	0.0105566	0.0336517	0	0	0.1207758	0	0	0	0	0	0	0	0.0695994	0	0.0315919	0	0	0.0793021
0.3618317	0.0598649	0.4709184	0	0.4498478	0.8751012	0.0105566	0.2459165	0	0	0.2252932	0.0412047	0.2214536	1.4028145	0	0	0	2.179132	0	0.1492344	0.3475114	0.0383391	0.2959553	0.4758128
0.9363616	0.5708544	12.63995	2.3116683	0.6786497	4.4519932	0	0	0.5304607	2.0394482	5.1701312	0.1947857	0.7002151	0.5735755	0	0	0	2.0898738	1.5479876	0.5255645	2.6020276	0.1033488	4.0955427	2.0324845
0.2982666	0.3142906	0.0218307	0.1627626	0.1434859	0.2474579	0.2412945	0.5410163	0.0938507	0.1675906	0.3739403	0.8428229	0.3332349	0.1000422	0.2013634	1.9787919	0.6118213	0.7694675	0.1679987	0.4563544	0.7869267	0.2033638	0.0418523	0.1497929
0.2347016	0.0427606	0	0	0.025207	0	0.2141489	0.5487821	0	0	0	0	0	0.0755875	0.0713162	0.097835	0.2643942	0.3724223	0	0.0886755	0.0717999	0.140021	0	0
0.3324939	0.4062259	0	0.0967777	0.063987	0.3711869	0.0271456	0.3313401	0.1101726	0.154699	0.1230984	0.3933174	0.2467626	0	0	0	0.0437015	0	0	0	0	0	0	0
0.3056011	0.2437356	0.0436613	0.0483889	0.031024	0.0989832	0.0588155	0.1346069	0.2999143	0.0850844	0.2392289	0.2678304	0.2362172	0.0200084	0.9187205	0.3881841	0.1901016	0.1108033	0.5519956	0.0519076	0.1522157	2.3203481	0.5381005	0.126296
6.4665183	0.1240058	0.8233276	1.4472671	0.2830939	1.052821	0	0	0.6528747	0.8095913	0.05342	0	0	1.20273	0	0	0	0	0.0767994	1.6437408	2.9437951	0	0	0
0.4620688	0.1689045	0.0592546	0.0659848	0.065926	0.1754702	0.0060324	0	0.4162076	0.0232048	0.0789688	0.0374588	0.0231999	0	0	0.3376886	0	0	0.3119975	0	0.0746719	0.6034238	0.1763774	0.2173466
0.0391169	0.1453861	0	0	0	0.0224962	0.0226214	0.0155316	0	0.0464097	0.0325165	0.0224753	0.0548361	0	0.0230729	0	0	0	0	0.0173025	0.0689279	0	0	0
0.6527639	0.1838707	0	0.004399	0	0	0	0	0	0.0103133	0.0139357	0.2135151	0.1729447	0	0	0	0	0	0	0	0	0.0133353	0	0
0	0	0.074848	0	0.1396079	0	0	0	0	0	0	0	0	0.0266779	0	0	0.0174806	0	0.6479948	0	0.1637037	0	0	0
0	0	0.0405426	0	0	0	0	0	0	0	0	0	0	0	0	0	0	0	0	0	0.022976	0	0	0.0293712
0	0	0	0	0	0	0.0150809	0	0	0	0	0	0	0	0	0	0	0	0	0	0	5.9275558	0	0
0	0	0	0	0.4731158	0.0967336	0	0	0.4508916	0.6265309	0.5109743	0	0	0	0	2.1334343	0	0	0	5.1193875	0	0	0	0
0	0	0	0	0.3722878	1.1360569	0	0.1346069	0	0	0.0743235	1.3953401	0.670688	0	0	0.4197437	0	0	1.4039888	0	0.591631	0	0	0
0	0	0	0	0	0	0.0226214	0	0	0	0.0139357	0	0	0	0	0	0	0	0	0	0	0	0	0
0	0	0	0	0	0	0	0	0	0	0	0	0	0	0	0	0	0	0	0	0	0.1016819	0	0
4.7918246	5.9052425	0	0	0	0	0.0271456	0	0.0836496	0.1108676	0	0	0	0	0.1929733	0	0.2993554	2.1421976	0	0	0	0.8351253	0	0
1.3813168	1.9114	0.6829877	2.8791378	0	0.0607397	0	0	0	0	0	0	0	0.9026033	0	0	0.1005135	0	0	0	0	0.9918154	2.7712176	3.3718096
0	0	0	0	0	0	0	0	0	0	0	0	0	0	0	0	0	0	0	0.0173025	0	0	0.0358734	0
0	0	0	0	0	0	0	0	0	0	0	0	0	0	0	0	0	0	0	0	0	0	0	0
1.5353397	0.7141025	0	0.0461894	0	0	0	0	0	0.2165786	0.0743235	0.0486964	0	0.0511327	0	0	0	0	0	0	0	0.0466737	0.2092613	0.0381825
0	0	0	0	0	0	0	0	0	0.0077349	0	0	0	0	0	0	0	0	0	0	0	0	0	0.0058742

TOC992.T0	TOC992.T3	TOC994.T0	TOC998.T0	TOC998.T3	TOC.CTRL1(	TOC.CTRL1(	TOC.CTRL1(	TOC.CTRL1(	TOC.CTRL1(	TOC.CTRL1(	TOC.CTRL1(	TOC.CTRL1(	TOC.CTRL1(	TOC.CTRL1(	TOC.CTRL11	TOC.CTRL11	TOC.CTRL11	TOC.CTRL11	TOC.CTRL11	TOC.CTRL11	TOC.CTRL11	TOC.CTRL11	TOC.CTRL11
0.3647026	0.1207828	0.0950302	0.1799469	0.4467663	0	0	0.1635338	0	0	0	0	0.6932486	0	0	0.3432412	0	0	0	0.0304675	0.3607567	0.0324733	0	0.1769513
0.1905984	0.0450377	0	0.046084	0.2017654	0.0231275	0	0	0	0.0423549	0	0	0	0	0	0	0	0.0326707	0	0	0	0	0.1551936	0.0933016
0.4398424	0.4524238	0.1797868	0	0.1513241	0	0.1687915	0	0.0179767	0	0.0076415	0	0.0375326	0	0	0	0	0.0343903	0	0.0209464	0	0.0280451	0.0196222	0
0	0	0	0	0	0.1172895	0	0	0	0	0	0	0.3775334	0	0	0	0.1893979	0.0085976	0.2171046	0.9673427	0	0.0265691	0.0927594	0
0.6707596	0.159679	0.2825222	0.1777524	0.1225005	0.0561668	0.0953175	0	0.3460519	0.1429479	0.2017362	0	0	0	0	0.169237	0	0.0240732	0.26783	0	0.164247	0.025093	0.2622237	0
1.7373774	1.5271864	4.6385001	1.2442669	0.9079445	1.2274094	1.6541562	0.2156489	2.945935	0.5823803	3.7122509	0.3155163	0.1523381	0.4866534	0.1289583	0.2216766	0.274731	1.0712566	0.2536269	0	0	1.163134	1.3628499	4.2757866
0	0	0.2003339	0	0	0	0	0	0	0	0	0	0.0971431	0	0	0.0738922	0.0291381	0	0	0	0	0	0.1159493	0
0	0	0.7448311	0.5464241	0.2486039	0	0	0	0.2157206	0.071474	0.1467172	0	0	0.210641	0.0573148	0.0834267	0	0.2390123	0.2434818	0	0.1055873	0.3203047	0	0
1.4624759	0.7349329	0.4263516	0.0921679	0.63412	0.6426141	0.4130426	0.0790713	0.4718889	0.1323592	0.7167747	0.2532434	1.4262375	0.2179045	0.0859722	0.0429052	1.190501	0.1134879	0.1927564	2.0394173	0.0469277	0.125465	1.9978951	0.4825944
0	0	0	0	0	0	0	0.0179707	0	0	0	0	0	0	0.0061409	0.0047672	0	0	0	0	0	0	0	0
0.0293228	0.024566	0	0.0087779	0	0	0	0	0	0	0	0	0	0	0	0	0	0	0.0263772	0.026659	0	0.0059042	0.032109	0
0.2547421	0.0757452	0.1797868	0.0153613	0	0.0115637	0	0	0.0292122	0.0979458	0	0	0.0176624	0	0	0	0	0	0.0121741	0.043797	0	0	0.1783835	0.0450421
1.4936314	0.5609237	0	0	0	0	0	0	0.3078513	0.2753071	0.5517178	0	0	0	0	0	0	0.0154756	0	0	0	0	0	0
0.3427105	0.1862922	0.9271863	0.1097237	0.0972798	0.3469125	0.3772985	0.2587787	0.0112355	0.4103134	0	0.1349248	0.1898706	0.1271109	0.1617096	0.2931852	0.0499511	0	0.3713097	0.2894411	0	0.2627384	0.7224531	0.8718873
0	0.0163773	0.120714	0.0109724	0	0	0	0	0.0764011	0.0158831	0.0519623	0	0.6292224	0	0	0	0	0.0257927	0.0933347	0	0.0293298	0.2465017	0.3193064	0
1.9811234	0.9191779	0	0	0	0	2.2896064	60.609927	0	0	0	0	0	0	20.633329	1.153672	0.239349	34.476236	0.6127625	0	24.00352	49.228021	0	28.283251
0	0	0	6.2103623	0.2954423	0	0	0.2192431	0	0	0	0	0	0	0	0	0	0.589793	3.0719286	0	0.9737498	0	0	0.6338073
0	0	0	0	0	0	0	0.0772742	0	0	0	0	0	0	0.0655026	0	0	0.003439	0	0	0.6129931	0.0118085	0	0
0.6231101	0.4565182	0	0.8273168	0.4972077	0.4229028	0.1687915	1.3657765	1.2516291	0	0.8604353	0	0	0.2959869	0.0266104	0.2312111	0	0.1048903	0.2820331	1.7633057	0.4194163	0	0.5761787	0
0.1741043	0.1944808	0	0.6276197	0	0	0	0	0	0.2753071	0.1176794	0.1037883	0.5166247	0.6682404	0	0.1835387	0.0499511	0.0171951	0.0750736	0.0799771	0	0.1461298	0.3639023	0
0	0	0.1284192	0.2457811	0	0	0	0	0	0	0	0	0	0.0780824	0	0	0	0	0	0	0.2522364	0	0	0
5.0728489	1.6909597	0	3.4650749	5.3864169	1.3926058	6.4657055	0.555296	1.9841805	2.9833757	6.0536129	10.48054	2.6493575	3.1051389	1.9998772	0.8867066	6.510292	2.1648669	13.854114	5.1242502	1.7627218	1.7697939	1.7874026	0.3088604
0.062311	0.0552735	0	0.0658342	0.2954423	0.046255	0.2740379	0.0341444	0	0.1085345	0.0412642	0	0	0.1525331	0.0511739	0.0834267	0.0832518	0	0.0324642	0.0799771	0.0967884	0.0856114	0.0445959	0
0	0	0.154103	1.1433211	1.0772834	0.0759904	1.110052	1.1645042	0.125837	1.40036	0.4340384	1.6875973	0.163377	0.2760123	0.335701	0.5744524	0.8845505	0.3490611	0.8440702	0.1085404	0.8652295	0.6228966	0.0231899	1.1743131
1.4972968	1.2323944	0.187492	0	0	0	0	2.0989829	0	0	0	0	0	0	1.9220928	0	0	8.4049797	2.9684488	0	0	0	0	0.575896
0.3042243	0.2169997	0.0128419	0	0	0.74008	2.9806585	0.7152356	0	0.0238247	0	0	0.1390913	0	1.8361206	1.0964651	2.0355069	0.003439	0	1.2320289	0.1231852	0.4132963	0	0
0	0	0.0462309	0	0	0	0	0	0	0	0	0	0	0	0	0	0	0	0	0	0	0	0.0285414	0
0	0	0	0	0.0648532	0	0	0.048521	0	0	0	0	0	0	0	0	0.1082274	0.0240732	0	0	0.1437161	0	0.0731372	0.0675632
0	0.0409433	0	0.0592508	0	0.0132157	0	0	0	0.0555909	0.0320944	0	0.1214289	0.1888506	0	0.0786595	0.0374633	0	0.0466673	0	0.0703916	0.0428057	0.1159493	0
0.1832677	0.184245	0.0410941	0.8317058	0	1.0176099	1.8328766	3.7019732	0.4516651	1.6227234	2.4131923	3.1219512	0.6513004	0	1.578204	0.2097585	0.5244864	1.4461105	0.677691	1.7347425	0.3783546	0.2952117	0.8919174	0
0.4563365	0.810678	1.4896623	1.5734381	1.7618447	2.1772888	1.6362842	0.6828883	0.1415667	0.0079416	0.803888	0.8157758	3.3403983	3.7061921	0	1.1489047	2.2290674	2.5930257	0.4342092	0.7712082	1.4518258	0.4162485	0.1105978	1.9625507
0.3866948	0.2231412	0.7859253	0.3116154	0.8142677	0.3155252	0.1886493	0.0449269	0.5415487	0.1138289	0.5868688	0.0290607	0.3157151	0.0254222	0.1719444	0.1215646	0.1914792	0.1048903	0.2881201	0.1180615	0.307963	0.1623664	0.1819512	0.4954636
0.2144232	0.1330658	0.1284192	0.0570563	0	0.0578187	0.0992891	0	0.1910027	0.8841593	0.2491136	0.2470161	0.1611692	0	0	0	0	0.0309512	0.0385513	0	0	0	0	0.5823306
0	0	0.2876589	0.041695	0	0.0297354	0	0	0	0	0.0489057	0.1619097	0.2384422	0.0671872	0.0614087	0.0476724	0	0	0.0831896	0	0	0	0	0.434335
0.0604783	0.0388962	2.3834596	0.0724177	0.4827959	0.1701523	0.0814171	0	0.1393196	0	0.226189	0.0145304	0.3311697	0	0.049127	0.1358663	0.0999022	0.0515854	0.1075378	0.0323717	0.0586596	0.0619945	0.053515	0.1479956
3.185192	1.7298559	0.3518685	0.8602339	0	0.1205934	1.392033	0.1222011	2.1392297	1.5671326	1.4259078	0	0.1368835	0.3777011	1.1892821	1.3681977	0.0499511	0	0.7243583	1.3215272	1.9973603	0.1608904	1.3628499	0
0.0733071	0	0.120714	0.0482784	0.1261034	0.1338091	0.1787204	0	0.0269651	0	0.0168113	0	0.1655848	0.056292	0.14124	0.8867066	0.2206173	0.0962927	0.2171046	0.0628392	0.8446986	0.1121804	0.0695696	0.1383437
0.0366535	0.0040943	0.0616412	0	0	0.044603	0.0556019	0.0700859	0	0	0.0213963	0	0.0353248	0	0.0245635	0.1811551	0.0499511	0.0842561	0	0.0361801	0.0586596	0	0.0285414	0
0.0403189	0	0	0	0	0	0	0	0	0	0	0	0	0	0	0	0.0124878	0	0	0	0	0	0	0.0933016
0	0.0163773	0	0.0109724	0	0.0033039	0.0536161	0.097042	0	0.0450021	0	0.5355475	0	0.0453968	0	0.069125	0	0.2905977	0.0466673	0.2399314	0.0703916	0	0	0
0	0	0	0	0	0	0	0.700859	0	0	0	0.3466528	0	0	0	0	0	0.0533049	0	0.087594	0.0351958	0.0309972	0	0.0193038
0	0	0	0	0	0	0.2144644	0	0.2831333	0	0.2567551	0.9590036	0	0	0	0	0	0	0	0	0	0	0	0
0	0	1.6977013	0	0	0	0	0	0	0	0	0	0.0949353	0.225168	0	2.0356113	0	0	0	0.68171	0	0	0	0
0	0	0.0077051	0.8031776	0.3314718	0.1090296	0	0.4870072	0	0.8762177	0	0	0.3510399	0.0490285	0.3295601	0.0238362	0.9178513	0	0.8684184	0	0.6804517	0	0.6903441	0
0	0	0	0.0592508	0	0	0	0	0	0	0	0.1266217	0	0	0	0	0	0.0550244	0	0	0	0	0	0
0	0	0	0	0	0	0	0	0	0	0.0886416	0.8531396	0	0.0690031	0	0	0	0.0515854	0	0	0	0	0	0
0	0	0	0	0	0	0	0	0.9819783	0	0.507397	0.0332122	0	0	0	1.3157581	0	0	0	0.2075597	0	3.719667	0	4.1857023
1.4129937	0.8884704	0	0	0	0.7599035	1.9778387	0	0.0786482	0	1.2776623	0	0	0	2.4502078	0.0286034	1.5609715	2.5276842	0.1034798	0	1.7744537	0.0073803	0	0
0	0	0	0	0	0	0.0297867	0	0	0	0	0.0062273	0	0	0	0	0.0416259	0.003439	0	0	0	0	0	0
0	0	0	0	0	0.6260944	0.3475118	0.0880566	0	0	0	0	0	0	1.0910282	0	0.0645202	0	0	0	0.3842206	0.2715947	0	0.1608648
0	0.0143302	0	0	0.6485318	0.0743384	0.0198578	0	0	0	0	0	0	0	0.0921131	0.5148619	0.0770079	0	0	0	0.0909224	0.126941	0.0660019	0
0	0.0266132	0	0	0	0.0412991	0	0	0	0	0	0	0	0	0	0	0.0145691	0	0	0	0.0293298	0	0	0

<table>
<tr><th>TOC.CTRL11</th><th>TOC.CTRL12</th><th>TOC.CTRL12</th><th>TOC.CTRL12</th><th>TOC.CTRL12</th><th>TOC.CTRL12</th><th>TOC.CTRL12</th><th>TOC.CTRL12</th><th>TOC.CTRL12</th><th>TOC.CTRL91</th><th>TOC.CTRL92</th><th>TOC.CTRL93</th><th>TOC.CTRL97</th><th>TOC.CTRL99</th><th>LEFSE HIGHLIGTED</th></tr>
<tr><td>0</td><td>0</td><td>0</td><td>0</td><td>0</td><td>0</td><td>0.0594189</td><td>0</td><td>0.2825737</td><td>0.102702</td><td>0</td><td>0.30829</td><td>0.1676602</td><td>0</td><td>Bacteria | Bacteroidetes | Bacteroidia | Bacteroidales | Rikenellaceae | Alistipes | Alistipes finegoldii</td></tr>
<tr><td>0</td><td>0</td><td>0.0259507</td><td>0</td><td>0</td><td>0</td><td>0</td><td>0</td><td>0</td><td>0</td><td>0</td><td>0</td><td>0</td><td>0</td><td>Bacteria | Bacteroidetes | Bacteroidia | Bacteroidales | Rikenellaceae | Alistipes | Alistipes ihumii</td></tr>
<tr><td>0</td><td>0</td><td>0</td><td>0</td><td>0</td><td>0</td><td>0</td><td>0</td><td>0.0395603</td><td>0</td><td>0</td><td>0.0392045</td><td>0</td><td>0</td><td>Bacteria | Bacteroidetes | Bacteroidia | Bacteroidales | Rikenellaceae | Alistipes | Alistipes indistinctus</td></tr>
<tr><td>0.0129391</td><td>0</td><td>0</td><td>0</td><td>0</td><td>0.1319641</td><td>0</td><td>0</td><td>0.4040804</td><td>0.0464604</td><td>0</td><td>0.0498966</td><td>0</td><td>0.0394756</td><td>Bacteria | Bacteroidetes | Bacteroidia | Bacteroidales | Rikenellaceae | Alistipes | Alistipes inops</td></tr>
<tr><td>0.0345043</td><td>0.0833442</td><td>0.0613381</td><td>0</td><td>0.0415191</td><td>0.1522662</td><td>0.0831864</td><td>0</td><td>0</td><td>0.0904756</td><td>0</td><td>0.074845</td><td>0</td><td>0.024932</td><td>Bacteria | Bacteroidetes | Bacteroidia | Bacteroidales | Rikenellaceae | Alistipes | Alistipes obesi</td></tr>
<tr><td>0.3795476</td><td>0.216174</td><td>0.0849297</td><td>0.3900971</td><td>0.3785566</td><td>0.807011</td><td>2.2856464</td><td>0</td><td>0.4860267</td><td>0.9414354</td><td>0</td><td>1.0531756</td><td>0.691931</td><td>0.4176103</td><td>Bacteria | Bacteroidetes | Bacteroidia | Bacteroidales | Rikenellaceae | Alistipes | Alistipes putredinis</td></tr>
<tr><td>0</td><td>0</td><td>0</td><td>0</td><td>0</td><td>0</td><td>0</td><td>0</td><td>0</td><td>0</td><td>0</td><td>0</td><td>0</td><td>0</td><td>Bacteria | Bacteroidetes | Bacteroidia | Bacteroidales | Rikenellaceae | Alistipes | Alistipes senegalensis</td></tr>
<tr><td>0</td><td>0</td><td>0</td><td>0</td><td>0</td><td>0.1861032</td><td>0.1703341</td><td>0</td><td>0.4097318</td><td>0</td><td>0</td><td>0.2869057</td><td>0</td><td>0</td><td>Bacteria | Bacteroidetes | Bacteroidia | Bacteroidales | Rikenellaceae | Alistipes | Alistipes shahii</td></tr>
<tr><td>0</td><td>0.0807397</td><td>0.0094366</td><td>0.0860057</td><td>0.1538649</td><td>0.3705145</td><td>0.0871477</td><td>0.0118725</td><td>0.2232332</td><td>1.3889229</td><td>0</td><td>0.2227529</td><td>0.263466</td><td>1.5312377</td><td>Bacteria | Bacteroidetes | Bacteroidia | Bacteroidales | Rikenellaceae | Alistipes | Alistipes unclassified.S187</td></tr>
<tr><td>0</td><td>0.005209</td><td>0</td><td>0</td><td>0</td><td>0</td><td>0</td><td>0</td><td>0</td><td>0</td><td>0</td><td>0</td><td>0</td><td>0</td><td>Bacteria | Bacteroidetes | Bacteroidia | Bacteroidales | Rikenellaceae | unclassified.G52 | unclassified.S192</td></tr>
<tr><td>0</td><td>0</td><td>0</td><td>0</td><td>0.0415191</td><td>0</td><td>0</td><td>0</td><td>0</td><td>0.0489057</td><td>0</td><td>0</td><td>0</td><td>0.0207766</td><td>Bacteria | Bacteroidetes | Bacteroidia | Bacteroidales | Barnesiellaceae | Coprobacter | Coprobacter secundus</td></tr>
<tr><td>0</td><td>0</td><td>0.0377465</td><td>0.0337879</td><td>0</td><td>0.1539581</td><td>0.0336707</td><td>0.0474901</td><td>0</td><td>0</td><td>0</td><td>0.1461259</td><td>0</td><td>0</td><td>Bacteria | Bacteroidetes | Bacteroidia | Bacteroidales | Barnesiellaceae | Coprobacter | Coprobacter unclassified.S178</td></tr>
<tr><td>0</td><td>0</td><td>0</td><td>0</td><td>0</td><td>0</td><td>0</td><td>0</td><td>0</td><td>0</td><td>0.0643182</td><td>0</td><td>0</td><td>0</td><td>Bacteria | Bacteroidetes | Bacteroidia | Bacteroidales | Barnesiellaceae | Barnesiella | Barnesiella intestinihominis</td></tr>
<tr><td>0.2178085</td><td>0.010418</td><td>0.044824</td><td>0.2918049</td><td>0.2051533</td><td>0.90683</td><td>0.28323</td><td>0.0379921</td><td>0.5764503</td><td>0.085585</td><td>0</td><td>0.229881</td><td>0.3220141</td><td>0</td><td>Bacteria | Bacteroidetes | Bacteroidia | Bacteroidales | Barnesiellaceae | Barnesiella | Barnesiella unclassified.S177</td></tr>
<tr><td>0.0711652</td><td>0.0468811</td><td>0.0401057</td><td>0</td><td>0</td><td>0</td><td>0.1029927</td><td>0</td><td>0</td><td>0</td><td>0</td><td>0.2477012</td><td>0</td><td>0.0727182</td><td>Bacteria | Bacteroidetes | Bacteroidia | Bacteroidales | Barnesiellaceae | unclassified.G51 | unclassified.S179</td></tr>
<tr><td>3.3900498</td><td>0</td><td>0.3043314</td><td>78.040914</td><td>0</td><td>0</td><td>0</td><td>0</td><td>0</td><td>12.603008</td><td>74.032753</td><td>0</td><td>0</td><td>16.301344</td><td>Bacteria | Bacteroidetes | Bacteroidia | Bacteroidales | Prevotellaceae | Prevotella | Prevotella unclassified.S33</td></tr>
<tr><td>1.2917556</td><td>0.1692929</td><td>0.0707747</td><td>0</td><td>0</td><td>0</td><td>0</td><td>0</td><td>2.9133347</td><td>0.8827485</td><td>0</td><td>0</td><td>0</td><td>0</td><td>Bacteria | Bacteroidetes | Bacteroidia | Bacteroidales | Prevotellaceae | Prevotellaceae_NK3B31_group | Prevotellaceae_N</td></tr>
<tr><td>0.0797912</td><td>0</td><td>0.1439087</td><td>0</td><td>0</td><td>0</td><td>0</td><td>0</td><td>0</td><td>0.0831397</td><td>0.0074213</td><td>0</td><td>0</td><td>0</td><td>Bacteria | Bacteroidetes | Bacteroidia | Bacteroidales | Prevotellaceae | unclassified.G7 | unclassified.S35</td></tr>
<tr><td>0.7698777</td><td>0</td><td>0.0754931</td><td>0.5436786</td><td>1.7999756</td><td>0</td><td>0</td><td>0.0831078</td><td>0.3503914</td><td>0.2176305</td><td>0.0494756</td><td>0.4098653</td><td>0</td><td>0</td><td>Bacteria | Bacteroidetes | Bacteroidia | Bacteroidales | Prevotellaceae | Paraprevotella | Paraprevotella unclassified.S182</td></tr>
<tr><td>0.0474434</td><td>0.0520901</td><td>0</td><td>0</td><td>0</td><td>0.0744413</td><td>0</td><td>0</td><td>0.1582413</td><td>0.0635775</td><td>0</td><td>0.1496899</td><td>0.1357249</td><td>0</td><td>Bacteria | Firmicutes | Clostridia | Clostridiales | Lachnospiraceae | Anaerostipes | Anaerostipes hadrus</td></tr>
<tr><td>0</td><td>0</td><td>0</td><td>0</td><td>0.3174991</td><td>0</td><td>0</td><td>0</td><td>0</td><td>0</td><td>0</td><td>0</td><td>0</td><td>0.0914172</td><td>Bacteria | Firmicutes | Clostridia | Clostridiales | Lachnospiraceae | Anaerostipes | Anaerostipes unclassified.S220</td></tr>
<tr><td>0.5261909</td><td>10.740982</td><td>1.2692271</td><td>0.540607</td><td>1.7047259</td><td>4.9960242</td><td>9.3525322</td><td>0</td><td>3.8684337</td><td>6.7954518</td><td>3.4781318</td><td>15.785159</td><td>5.5567383</td><td>2.5846128</td><td>Bacteria | Firmicutes | Clostridia | Clostridiales | Lachnospiraceae | Agathobacter | Agathobacter unclassified.S70</td></tr>
<tr><td>0.1358608</td><td>0</td><td>0.2099651</td><td>0</td><td>0.1099035</td><td>0.2402423</td><td>0.0495157</td><td>0</td><td>0.1215067</td><td>0</td><td>0</td><td>0</td><td>0</td><td>0.0664852</td><td>Bacteria | Firmicutes | Clostridia | Clostridiales | Lachnospiraceae | Coprococcus | Coprococcus catus</td></tr>
<tr><td>0.3881736</td><td>0.3281677</td><td>0.3350005</td><td>0.0522177</td><td>0.1318842</td><td>0.143807</td><td>0.0435738</td><td>0.1994586</td><td>0.5481929</td><td>0</td><td>0.2152187</td><td>0</td><td>0.7265276</td><td>0.6669298</td><td>Bacteria | Firmicutes | Clostridia | Clostridiales | Lachnospiraceae | Coprococcus | Coprococcus comes</td></tr>
<tr><td>0.2091825</td><td>1.4246647</td><td>1.4414457</td><td>0</td><td>0</td><td>1.8305784</td><td>0</td><td>0</td><td>4.8659188</td><td>0</td><td>0</td><td>0</td><td>0.2182244</td><td>5.1442937</td><td>Bacteria | Firmicutes | Clostridia | Clostridiales | Lachnospiraceae | Coprococcus | Coprococcus eutactus</td></tr>
<tr><td>6.9159604</td><td>2.4013543</td><td>1.833066</td><td>0</td><td>0</td><td>0.7900925</td><td>0</td><td>0</td><td>1.3507022</td><td>0.0366793</td><td>0.9820898</td><td>0.0178202</td><td>0</td><td>3.434377</td><td>Bacteria | Firmicutes | Clostridia | Clostridiales | Lachnospiraceae | Coprococcus | Coprococcus unclassified.S223</td></tr>
<tr><td>0</td><td>0</td><td>0.103803</td><td>0</td><td>0</td><td>0</td><td>0</td><td>0.0332431</td><td>0</td><td>0</td><td>0</td><td>0</td><td>0</td><td>0</td><td>Bacteria | Firmicutes | Clostridia | Clostridiales | Lachnospiraceae | Hungatella | Hungatella hathewayi</td></tr>
<tr><td>0.0948869</td><td>0</td><td>0.0636973</td><td>0</td><td>0.0537306</td><td>0</td><td>0</td><td>0</td><td>0</td><td>0</td><td>0.0494756</td><td>0.078409</td><td>0</td><td>0.0540192</td><td>Bacteria | Firmicutes | Clostridia | Clostridiales | Lachnospiraceae | Howardella | Howardella unclassified.S74</td></tr>
<tr><td>0.107826</td><td>0</td><td>0</td><td>0</td><td>0.0903651</td><td>0.0930516</td><td>0</td><td>0</td><td>0.1780214</td><td>0.0904756</td><td>0</td><td>0.0392045</td><td>0.0266127</td><td>0.2451642</td><td>Bacteria | Firmicutes | Clostridia | Clostridiales | Lachnospiraceae | Lachnospiraceae_FCS020_group | Lachnospiraceae_FCS</td></tr>
<tr><td>0.6167648</td><td>1.7189738</td><td>0.3114089</td><td>0.2211574</td><td>0.7253633</td><td>3.405688</td><td>3.4819466</td><td>2.270029</td><td>0.0678177</td><td>0.4719403</td><td>1.481793</td><td>0.5203507</td><td>3.8934426</td><td>2.0693524</td><td>Bacteria | Firmicutes | Clostridia | Clostridiales | Lachnospiraceae | Lachnospira | Lachnospira pectinoschiza</td></tr>
<tr><td>0.8992689</td><td>3.826019</td><td>0.5921487</td><td>0</td><td>0.4518256</td><td>0.9592772</td><td>3.0858207</td><td>0</td><td>1.7321767</td><td>0.8289522</td><td>1.4768454</td><td>0.0694989</td><td>1.2055567</td><td>1.5208493</td><td>Bacteria | Firmicutes | Clostridia | Clostridiales | Lachnospiraceae | Lachnospira | Lachnospira unclassified.S233</td></tr>
<tr><td>0.4399301</td><td>0.0338586</td><td>0.058979</td><td>0.0491461</td><td>0.1050189</td><td>0.331602</td><td>0.1168571</td><td>0</td><td>0.0932493</td><td>0.1271549</td><td>0.0643182</td><td>0.5078765</td><td>0.1836278</td><td>0.0893395</td><td>Bacteria | Firmicutes | Clostridia | Clostridiales | Ruminococcaceae | Oscillibacter | Oscillibacter unclassified.S270</td></tr>
<tr><td>0.0452869</td><td>0</td><td>0</td><td>0</td><td>0.0830382</td><td>0</td><td>0</td><td>0.3822957</td><td>0</td><td>0</td><td>0</td><td>0.1176135</td><td>0.1516926</td><td>0</td><td>Bacteria | Firmicutes | Clostridia | Clostridiales | Ruminococcaceae | Flavonifractor | Flavonifractor plautii</td></tr>
<tr><td>0.0517565</td><td>0</td><td>0.0377465</td><td>0.0368596</td><td>0</td><td>0.0812087</td><td>0.1029927</td><td>0</td><td>0</td><td>0.0611322</td><td>0</td><td>0.0249483</td><td>0</td><td>0</td><td>Bacteria | Firmicutes | Clostridia | Clostridiales | Ruminococcaceae | Flavonifractor | Flavonifractor unclassified.S265</td></tr>
<tr><td>0.3256346</td><td>0</td><td>0.3350005</td><td>0.0337879</td><td>0</td><td>0.0287614</td><td>0.015845</td><td>0</td><td>0.4379892</td><td>0.0415699</td><td>0</td><td>0.1140495</td><td>0</td><td>0.0290873</td><td>Bacteria | Firmicutes | Clostridia | Clostridiales | Ruminococcaceae | UBA1819 | UBA1819 unclassified.S287</td></tr>
<tr><td>0</td><td>0.4219299</td><td>1.5145796</td><td>0.7586927</td><td>0.9207473</td><td>0.8053192</td><td>0.3129395</td><td>0</td><td>1.8565091</td><td>0.102702</td><td>0.1632693</td><td>1.1583149</td><td>0.3779008</td><td>0.6877065</td><td>Bacteria | Firmicutes | Clostridia | Clostridiales | Ruminococcaceae | Ruminococcaceae_UCG-002 | Ruminococcaceae_UCG-</td></tr>
<tr><td>0.0776347</td><td>0.1172028</td><td>0.3916203</td><td>0.0153582</td><td>0.0610575</td><td>0.0845923</td><td>0.0792252</td><td>0</td><td>0.161067</td><td>0.1393813</td><td>0.0197902</td><td>0.1015753</td><td>0</td><td>0.0997278</td><td>Bacteria | Firmicutes | Clostridia | Clostridiales | Family_XIII | Family_XIII_AD3011_group | Family_XIII_AD3011_group uncl</td></tr>
<tr><td>0.1487999</td><td>0</td><td>0.0825705</td><td>0</td><td>0.1880571</td><td>0.0186103</td><td>0</td><td>0</td><td>0</td><td>0.0268982</td><td>0</td><td>0.0463326</td><td>0.0239515</td><td>0.1288151</td><td>Bacteria | Firmicutes | Clostridia | Clostridiales | Family_XIII | Family_XIII_UCG-001 | Family_XIII_UCG-001 unclassified.S64</td></tr>
<tr><td>0.0172522</td><td>0</td><td>0</td><td>0</td><td>0</td><td>0</td><td>0</td><td>0</td><td>0</td><td>0</td><td>0</td><td>0</td><td>0</td><td>0</td><td>Bacteria | Firmicutes | Clostridia | Clostridiales | Family_XIII | unclassified.G20 | unclassified.S66</td></tr>
<tr><td>0</td><td>0.4740201</td><td>0.0330282</td><td>0</td><td>0.0757113</td><td>0.087976</td><td>0</td><td>0</td><td>0</td><td>0</td><td>0.2102711</td><td>0</td><td>0.2900788</td><td>0.0457086</td><td>Bacteria | Firmicutes | Clostridia | Clostridiales | Clostridiaceae_1 | Clostridium | Clostridium unclassified.S211</td></tr>
<tr><td>0.0431304</td><td>0</td><td>0</td><td>0.0552893</td><td>0.4054219</td><td>0</td><td>0</td><td>0.0712352</td><td>0</td><td>0</td><td>0.0519493</td><td>0</td><td>0.1809666</td><td>0.1059608</td><td>Bacteria | Firmicutes | Clostridia | Clostridiales | Peptostreptococcaceae | Romboutsia | Romboutsia unclassified.S253</td></tr>
<tr><td>0</td><td>0</td><td>0</td><td>0</td><td>0</td><td>0</td><td>1.8915011</td><td>0</td><td>0</td><td>0</td><td>0.0915298</td><td>0</td><td>1.4264424</td><td>0</td><td>Bacteria | Firmicutes | Negativicutes | Selenomonadales | Acidaminococcaceae | Acidaminococcus | Acidaminococcus intes</td></tr>
<tr><td>0</td><td>0</td><td>0.0731339</td><td>0</td><td>0</td><td>0</td><td>0.037632</td><td>0</td><td>0</td><td>0.0660227</td><td>0</td><td>1.4648229</td><td>0</td><td>0</td><td>Bacteria | Firmicutes | Negativicutes | Selenomonadales | Acidaminococcaceae | Phascolarctobacterium | Phascolarctobac</td></tr>
<tr><td>0</td><td>0.1588749</td><td>0</td><td>0.7249048</td><td>0</td><td>0.763023</td><td>0</td><td>0</td><td>0.4803753</td><td>1.0857073</td><td>0</td><td>0</td><td>0</td><td>0.2659409</td><td>Bacteria | Firmicutes | Negativicutes | Selenomonadales | Acidaminococcaceae | Phascolarctobacterium | Phascolarctobac</td></tr>
<tr><td>0</td><td>0</td><td>0</td><td>0</td><td>0</td><td>0</td><td>0</td><td>0</td><td>0</td><td>0</td><td>0</td><td>0</td><td>0</td><td>0</td><td>Bacteria | Firmicutes | Negativicutes | Selenomonadales | Veillonellaceae | Allisonella | Allisonella unclassified.S311</td></tr>
<tr><td>0</td><td>0</td><td>0</td><td>0</td><td>0</td><td>0</td><td>0</td><td>0</td><td>0.0056515</td><td>0</td><td>0</td><td>0</td><td>0</td><td>0</td><td>Bacteria | Firmicutes | Negativicutes | Selenomonadales | Veillonellaceae | Veillonella | Veillonella unclassified.S104</td></tr>
<tr><td>0</td><td>0</td><td>0</td><td>0</td><td>0</td><td>0</td><td>1.0834043</td><td>0</td><td>0</td><td>0</td><td>0</td><td>0</td><td>0</td><td>0</td><td>Bacteria | Firmicutes | Negativicutes | Selenomonadales | Veillonellaceae | Dialister | Dialister invisus</td></tr>
<tr><td>2.8703284</td><td>1.3230889</td><td>1.9179957</td><td>2.7890404</td><td>1.5215533</td><td>0</td><td>0.0079225</td><td>0</td><td>0</td><td>0.102702</td><td>2.3500891</td><td>0</td><td>1.5834575</td><td>1.7826349</td><td>Bacteria | Firmicutes | Negativicutes | Selenomonadales | Veillonellaceae | Dialister | Dialister unclassified.S314</td></tr>
<tr><td>0</td><td>0</td><td>0</td><td>0.0092149</td><td>0</td><td>0</td><td>0</td><td>0.004749</td><td>0</td><td>0</td><td>0</td><td>0</td><td>0.0372578</td><td>0</td><td>Bacteria | Firmicutes | Negativicutes | Selenomonadales | Veillonellaceae | unclassified.G24 | unclassified.S103</td></tr>
<tr><td>0</td><td>0.1119937</td><td>0.2925356</td><td>0</td><td>0</td><td>0</td><td>0</td><td>0.1021038</td><td>0.2938766</td><td>0</td><td>0.0247378</td><td>0</td><td>0</td><td>0</td><td>Bacteria | Firmicutes | Erysipelotrichia | Erysipelotrichales | Erysipelotrichaceae | Catenibacterium | Catenibacterium uncla</td></tr>
<tr><td>0.1531129</td><td>0</td><td>0.2264792</td><td>0.0276447</td><td>0</td><td>0.0845923</td><td>0</td><td>0</td><td>0.0847721</td><td>0.1516078</td><td>0</td><td>0</td><td>0</td><td>0.1433588</td><td>Bacteria | Tenericutes | Mollicutes | Mollicutes_RF39 | unclassified.F36 | unclassified.G77 | unclassified.S358</td></tr>
<tr><td>0.0280348</td><td>0.0520901</td><td>0</td><td>0</td><td>0</td><td>0.0456799</td><td>0</td><td>0</td><td>0.1158552</td><td>0</td><td>0</td><td>0.2993799</td><td>0</td><td>0.0415533</td><td>Bacteria | Lentisphaerae | Lentisphaeria | Victivallales | vadinBE97 | unclassified.G66 | unclassified.S318</td></tr>
</table>

K3B31_group unclassified.S184

5020_group unclassified.S235

-002 bacterium
assified.S217

stini
terium faecium
terium unclassified.S308

ssified.S293

Supplementary Table 6. Individual abundace for top taxa in oropharyngeal samples

LEFSE HIGHLIGHTED	TOC1000.T	TOC1000.T
Bacteria | Actinobacteria | Actinobacteria | Actinomycetales | Actinomycetaceae | Actinomyces | Actinomyces odontolyticus	0	0
Bacteria | Actinobacteria | Actinobacteria | Actinomycetales | Actinomycetaceae | Actinomyces | Actinomyces unclassified.S2	2.596491	10.5825
Bacteria | Actinobacteria | Actinobacteria | Corynebacteriales | Corynebacteriaceae | Corynebacterium | Corynebacterium unclassified.S7	0	0
Bacteria | Actinobacteria | Actinobacteria | Micrococcales | Micrococcaceae | Rothia | Rothia unclassified.S18	3.221691	5.088801
Bacteria | Actinobacteria | Actinobacteria | Micrococcales | unclassified.F2 | unclassified.G3 | unclassified.S19	0	0
Bacteria | Actinobacteria | Actinobacteria | Micrococcales | Micrococcaceae | Rothia | Rothia dentocariosa	0	0
Bacteria | Actinobacteria | Actinobacteria | Micrococcales | Micrococcaceae | Rothia | Rothia mucilaginosa	0.797448	0
Bacteria | Actinobacteria | Actinobacteria | Propionibacteriales | Propionibacteriaceae | Cutibacterium | Cutibacterium unclassified.S21	0	0.547529
Bacteria | Actinobacteria | Actinobacteria | unclassified.O2 | unclassified.F3 | unclassified.G4 | unclassified.S22	0	0
Bacteria | Actinobacteria | Coriobacteriia | Coriobacteriales | Atopobiaceae | Atopobium | Atopobium unclassified.S23	1.894737	4.306616
Bacteria | Actinobacteria | Coriobacteriia | Coriobacteriales | Atopobiaceae | Atopobium | Atopobium parvulum	0.77193	0
Bacteria | Firmicutes | Clostridia | Clostridiales | Lachnospiraceae | Oribacterium | Oribacterium asaccharolyticum	0	0
Bacteria | Firmicutes | Clostridia | Clostridiales | Lachnospiraceae | Oribacterium | Oribacterium parvum	0	0
Bacteria | Firmicutes | Clostridia | Clostridiales | Lachnospiraceae | Oribacterium | Oribacterium sinus	0.637959	3.041318
Bacteria | Firmicutes | Clostridia | Clostridiales | Lachnospiraceae | Oribacterium | Oribacterium unclassified.S81	0.267943	0
Bacteria | Firmicutes | Clostridia | Clostridiales | Lachnospiraceae | Catonella | Catonella morbi	0	0
Bacteria | Firmicutes | Clostridia | Clostridiales | Lachnospiraceae | Catonella | Catonella unclassified.S73	1.811802	0.395693
Bacteria | Firmicutes | Clostridia | Clostridiales | Lachnospiraceae | Stomatobaculum | Stomatobaculum unclassified.S83	0.267943	4.403239
Bacteria | Firmicutes | Clostridia | Clostridiales | Lachnospiraceae | Stomatobaculum | Stomatobaculum longum	0	5.659336
Bacteria | Firmicutes | Clostridia | Clostridiales | Lachnospiraceae | Lachnoanaerobaculum | Lachnoanaerobaculum unclassified.S76	1.594896	0
Bacteria | Firmicutes | Clostridia | Clostridiales | Lachnospiraceae | Lachnoanaerobaculum | Lachnoanaerobaculum cf.	0.478469	1.062851
Bacteria | Firmicutes | Clostridia | Clostridiales | Lachnospiraceae | Butyrivibrio | Butyrivibrio unclassified.S72	0	0
Bacteria | Firmicutes | Clostridia | Clostridiales | Lachnospiraceae | Johnsonella | Johnsonella unclassified.S75	0.248804	0
Bacteria | Firmicutes | Clostridia | Clostridiales | Lachnospiraceae | unclassified.G21 | unclassified.S84	0	0
Bacteria | Firmicutes | Clostridia | Clostridiales | Family_XIII | Mogibacterium | Mogibacterium unclassified.S65	0	0
Bacteria | Firmicutes | Clostridia | Clostridiales | Peptostreptococcaceae | unclassified.G22 | unclassified.S88	0	0
Bacteria | Bacteroidetes | Bacteroidia | Bacteroidales | unclassified.F4 | unclassified.G8 | unclassified.S37	0.076555	0
Bacteria | Fusobacteria | Fusobacteriia | Fusobacteriales | Leptotrichiaceae | Leptotrichia | Leptotrichia hongkongensis	0	0
Bacteria | Fusobacteria | Fusobacteriia | Fusobacteriales | Fusobacteriaceae | Fusobacterium | Fusobacterium nucleatum	30.63477	4.288212
Bacteria | Fusobacteria | Fusobacteriia | Fusobacteriales | Fusobacteriaceae | Fusobacterium | Fusobacterium periodonticum	4.15311	0
Bacteria | Fusobacteria | Fusobacteriia | Fusobacteriales | Fusobacteriaceae | Fusobacterium | Fusobacterium simiae	0	0
Bacteria | Fusobacteria | Fusobacteriia | Fusobacteriales | Fusobacteriaceae | Fusobacterium | Fusobacterium unclassified.S106	13.8118	1.463145
Bacteria | Fusobacteria | Fusobacteriia | Fusobacteriales | Fusobacteriaceae | unclassified.G26 | unclassified.S107	0	0
Bacteria | Fusobacteria | Fusobacteriia | Fusobacteriales | Leptotrichiaceae | Leptotrichia | Leptotrichia unclassified.S108	1.154705	2.070489
Bacteria | Fusobacteria | Fusobacteriia | Fusobacteriales | Leptotrichiaceae | Oceanivirga | Oceanivirga unclassified.S109	0	0
Bacteria | Fusobacteria | Fusobacteriia | Fusobacteriales | unclassified.F11 | unclassified.G28 | unclassified.S113	0.657097	0
Bacteria | Fusobacteria | Fusobacteriia | Fusobacteriales | Leptotrichiaceae | Leptotrichia | Leptotrichia wadei	0	0

TOC1001.T	TOC1001.T	TOC1002.T	TOC1002.T	TOC1012.T	TOC1012.T	TOC1014.T	TOC1014.T	TOC1021.T	TOC1021.T	TOC1025.T	TOC1027.T	TOC1027.T	TOC1030.T	TOC1030.T	TOC1031.T	TOC1031.T
3.911098	0	14.40451	12.10044	0	0	32.80467	12.97689	1.355672	2.531439	0	10.24485	4.054836	6.683384	0	5.828162	15.53941
16.02684	19.81727	4.43133	7.410347	9.591723	30.55627	21.00723	6.851155	13.08351	11.10567	6.080831	11.92342	4.025914	16.98648	0.622222	4.075235	6.282517
0	0	0	0	0	0	0	0	0	0	0	0	0	0	0	1.503423	0
1.313321	0	0.053648	0	7.941834	0.11177	0	0	3.448864	0	0	0.790733	0.399121	0.294374	0	0.524599	1.462648
0.101025	0.005783	0.214592	0	0	0	0	0	0	0	0	0	0	0.054514	0	0	0.127614
4.394574	11.47863	0	3.066599	0	0	2.420701	0.779151	0	0	0.852799	0	0	0	0.492929	1.599386	0
17.8958	25.69826	11.12124	16.51634	8.668904	8.649299	0	3.774852	4.378224	5.797812	10.19651	0	0	18.58918	2.311111	0	24.38402
0	0.156133	0	0	0	0	0	0	0	0	0	0	0	0	0	0.262299	0
0.454611	0	0	0	0	0	0	0	0.170525	0	0	0	0.104118	0.196249	0	0	0
0	1.330018	2.773605	5.498232	0	0	0	0	0	0	0	0	0	0	0.09697	0	0
6.949055	13.23657	0	0	5.369128	25.59539	0	2.36432	3.035341	2.10681	0	6.533953	1.203147	9.648932	0.436364	2.194357	4.790419
2.273055	2.359336	1.223176	1.876037	0	0	7.679466	0	0	0	0	1.206909	0	0	0	1.420255	3.052911
0	0	0	0	0	0	0	0	0	0	0	1.581466	0.341277	0	0	0	0
0.808197	1.85046	1.845494	1.702865	7.074944	0.653426	0	3.049436	1.104148	0.70227	0.593252	1.921343	0.595789	2.191452	0	0	1.12889
0.721605	0	1.046137	0.461794	0	0	0	0	0.200367	0	0	0	0	0	0.589899	0	0
0	0.208177	0	0	0	0	0	0	0	0	0.951675	0	0	0	11.98384	4.024055	0
0.360802	0	0	0.562811	2.209172	0	0	1.598603	0.537153	0.979912	1.693239	1.484359	0.503239	0	0	1.375472	0.628252
3.398759	1.960331	2.070815	1.342088	0	0	0	9.470715	0	0.767598	0	3.558299	2.313744	0	0	1.14516	1.649161
0	0	2.237124	1.77502	1.454139	5.511134	0	0	3.286865	3.544014	0	0.769924	0.387552	6.966856	0	0.31348	3.740061
2.785395	0	2.76824	2.850133	1.985459	1.951681	0	0.806018	3.282602	1.878164	0	2.330582	6.177696	22.50327	0	1.330689	6.518111
0.216481	0	0.171674	0	0	0.576047	3.951029	3.600215	0.221682	0.293974	1.223582	1.317889	1.272559	0	0	0	0
0.389667	0	0.091202	0	0	0	0	0	0	0	1.446051	0.173406	0	0	1.00202	0	0
2.301919	0	0.69206	0	0	0	0	0	0.161999	0	0.939315	2.365263	1.856779	0	0.581818	0	0
0	0	0.187768	0	0	0	0	0	0.012789	0	0	0	0	0.806803	0	0.185529	0
0.122673	0	0	0	0	0.137563	0	0.268673	0.179051	0	0.234829	0	0	0.163541	0.169697	0	0
0	0	0.059013	0	0	0	0	0	0	0	0	0	0.231374	0	0	0.844476	0
0.173185	0	0	0	0	0	0	0	0.080999	0	0	0	0.295002	0.654165	0	0	0.451556
0	0.427919	0	0.317483	0	0	0	0	0.903781	0	0.457298	0	0	0	0	0.115156	0
0.902006	3.643093	13.73391	6.443466	0	1.281059	0	0.685116	8.394083	5.552834	14.15153	0	0	3.336241	9.422222	23.35743	1.649161
3.752345	0	2.859442	2.958366	12.02461	2.235405	10.62883	0	5.490898	15.92357	0.655049	15.47479	45.31467	0	0	0.80609	1.472465
0	0.312265	0	0	0	0	0	0	0	0	0	0	0	0	1.721212	0.02559	0
0.62058	6.025559	5.659871	4.192222	0	0.249334	0	12.88286	1.581617	10.33807	4.647139	0	1.833642	0.577846	24.38788	7.8114	0
0	0	0	0	0	0	0	0	0.029842	0	0	0	0.804026	0	0	0	0
9.517968	0.161915	7.891631	2.056425	10.45861	6.525664	1.057318	3.98979	28.00017	1.584191	0.865159	4.321287	7.074271	0.403402	0	2.680571	1.168155
0	0	0	0	0	0	0	0	0	0	20.39303	0	0	0	0	0	0
0.230914	0	0.429185	0	0	0	0	0	0.289892	1.649518	0	0.201151	0.416474	0.076319	0.145455	0	0
0	0.555138	0	1.089545	0	0	0	0	1.023149	0	0	0	0	1.013956	0	0	0

TOC2017.T	TOC2017.T	TOC2020.T	TOC2022.T	TOC2022.T	TOC2024.T	TOC2024.T	TOC2026.T	TOC2026.T	TOC2027.T	TOC2027.T	TOC2029.T	TOC2029.T	TOC2030.T	TOC2030.T	TOC2031.T	TOC2032.T
0	0	4.239619	0	0	0.399588	0.536249	14.781	9.773035	2.214773	15.6377	5.768274	0	0.581698	0	0	5.108557
1.304255	6.185434	3.863782	0	0	3.983522	4.305767	9.151179	6.156843	17.69558	8.532356	4.14719	2.510645	0.642473	4.488687	1.578947	7.360355
0	0	0	0.104373	0	0.906282	0	0.391262	0	0.331463	0	0	0	0	0	0	0
0	0	0.04125	27.86036	0	1.717817	5.325693	4.466906	0	1.548081	1.476563	0	1.9307	2.452683	0.012164	0	4.221281
0	0	0	0.03959	0	0	0	0	0	0	0	0	0	0	0	0	0.060496
3.99829	0	0	0.550657	11.49583	0	0.457389	0	0	10.23767	0	0	0	0.473172	0.843403	0	0
37.56682	33.03202	5.050875	63.64945	0	0	0.520477	5.868927	5.123645	4.915439	0.971162	2.018896	0	0	2.518044	10.47368	14.29724
0	0	0.132918	0	2.554629	0.201854	0	0.662971	0	0.199631	0	0	0	0	0.028384	0	0
0	0.025826	0	0.03959	0	0	0	0	0	0	0	0	0	0	0	0	0.194932
0	0	0	0.223142	0	0	0	0	0	0	0	0	0	1.454246	2.368016	0	0
0.427625	0.736054	3.465029	0	0	1.021627	2.549813	3.880013	3.971883	0.52356	6.60985	0	0.396418	1.34572	1.151569	5.684211	2.628218
0	0	0	0	0	1.239959	1.419484	0	0.533537	0	1.169359	0	0	0	0	0	0
0	0	3.093776	0	0	0	0	1.173785	0.79607	0.263663	0	0	0.82954	0	0.823129	0	0.322646
3.442378	2.453512	1.205427	0	0	0	1.119815	1.391153	0	4.422012	1.060351	0.358031	0	0	0	4.473684	2.910533
0	0	0.898341	0	0	0	0	1.34768	0	0	0	0	0	0.894252	0	0	0
0	1.097624	0	0.079179	0	0.523172	0	0	0	0	0	0	0	0	0	0	0
0	1.020145	3.414612	0	0	0	0.467904	2.619281	0.880759	0.154431	1.189178	0	0.462487	0.243098	0.23518	4.947368	3.179404
1.92431	0	0	0	0	0.061792	2.071395	0.565156	1.558266	1.156352	0.921613	0	1.108501	0	0.296002	1.263158	1.875378
0	1.252583	0.944175	0	0	0	0	0	1.287263	1.280651	3.924289	11.71556	0	0.408057	0.316276	0	0
0	0	1.260427	0	0	0	0.725514	0	2.39668	2.075408	5.985532	7.777225	2.084863	0.972391	1.248885	1.421053	0.746118
3.570665	0.72314	0	0	0	1.487127	0	2.184545	1.075542	1.126219	0	0	2.870357	0.256121	0	0	0.954494
0	0	0.270419	0	0	0	0	0.586893	0	0	0.267565	0	0	0.086821	0	0	0
1.496686	0	0.476671	0	0	0	0	0.108684	0	0.595126	0	1.402287	4.859786	0.724952	2.169329	0	0
0.042762	0	0.087084	0	0	0	0	0	0	0	0	0	0	0	0	0	0.201654
0	0	0.169585	0	0	0.140062	0.241838	0	0.279472	0.026366	0.158557	0	0	0.230075	0	0	0
0	0.167872	0	0	0	0	0	0	0	0	0	0	0	0	0	0	0
0.748343	0	0	0	0	0	0	0	0.008469	0	0	0	0	0.008682	0	0	0.295759
0	0	0	0.471477	0	0.350154	0	0	0	0	0.505401	0	0	0.160618	0	0	0
0	11.97056	1.466679	0.626237	47.74623	48.87333	25.2931	5.423324	2.896341	0.632792	2.804479	0.666335	0	36.3518	8.008272	3.842105	0.463803
0	8.910124	4.675039	0.57585	0	2.030896	12.12344	8.67297	18.05556	7.06618	10.10802	7.140726	25.87726	0.269144	6.540427	8.263158	2.964307
0	0	0	0	0	0	0	0	0	0	0	0	0	0.199687	0	1.157895	0
3.65619	1.239669	0.036667	0.658629	0.432809	1.136972	0.820146	3.1627	1.795393	0.018833	1.020712	1.034311	3.30348	5.495746	3.503366	14.73684	0
0	0	0	0	0	0	0	0	0	0	0	0	0.477169	0	0	0	0
0	3.228306	4.587955	0.194349	0	0.605561	12.19179	0.760787	4.225949	8.218765	15.20166	6.126305	18.56556	5.773572	4.139972	5.421053	7.75694
0	0	1.837932	0	0	0	0	0	0	0	0	0	0	2.700122	0.543346	0	0
0	0	0	0	0	0	0	0	0	0	0	0	0.323007	0	0	0	0.120992
0	0	0	0	0	0	0.378529	0.358657	0	0	0	0	0	0	0	0	0

TOC2032.T	TOC493.T0	TOC569.T0	TOC569.T3	TOC644.T0	TOC644.T3	TOC834.T0	TOC834.T3	TOC875.T0	TOC875.T3	TOC884.T0	TOC884.T3	TOC885.T0	TOC885.T3	TOC893.T3	TOC896.T0	TOC897.T0
4.704161	0	0	4.718519	1.543027	1.107151	6.849445	6.461728	4.192688	3.054895	0	0	3.413605	0	1.065417	0	0
2.529793	6.4264	1.27662	10.16296	10.87537	25.39622	1.90684	1.175878	1.375342	5.718017	2.988177	13.53885	13.04625	11.9704	3.696996	2.113035	15.99846
0	0	0	0	0	0	0	0.263173	0	0	0	0	0	0.311526	0	0	0
16.01505	0	0.283381	0.955556	0	0.007583	4.620055	1.44465	0	0.564339	5.352735	6.279301	1.701898	4.267913	4.639889	5.002546	1.512402
0.020907	0.008684	0.005612	0.022222	0	0	0	0	0.017543	0	0.006496	0	0	0.007788	0	0.038187	0
0	0	0	0	0	0	14.695	1.030293	0.378921	0.592323	1.565545	2.443529	0	0	2.343916	0	3.008304
5.080493	11.90621	1.139138	1.725926	2.522255	14.02897	14.66654	51.9346	0.494702	1.049391	14.79797	19.50561	0.848497	4.774143	8.390156	0	24.06644
0	0.156318	0	0	1.409496	0	0	0.604737	0	0	0	0	0	0	0	0	0
0	0	0	0	0	0	0	0	0	0	0	0	0	0	0	0	0
0	0	0	0	0	0.015166	1.090978	0	0.315767	5.279604	0	0.539849	0	0	0	0	0
0.292703	0.503691	0.600432	4.392593	21.21662	15.87928	2.561427	2.928495	1.592871	0.746234	0.669092	2.869726	21.42822	19.16667	3.563818	0.751018	1.38041
0	0	0.527482	0	10	1.819974	1.147899	0	0.533296	0.74157	0	0	1.898082	4.392523	0	0	0
0	0	0.440504	0	0	0	0	0	1.796365	0.443076	0	0	0	0	0.463456	0.623727	0
3.784236	0	1.329929	1.081481	0	0	0	1.327062	0.382429	0.657619	2.026764	3.452195	0	0	0	1.056517	0.736952
0	0	0	2.22963	0	0	0	0	0.501719	0.657619	1.110822	0	0	0.155763	0.34626	2.049389	0
0	0	0	0	0	0	0	0	0	0	0	0	0.176566	0	0	0	0
0.940832	0.738168	0.533094	0.807407	0	0.272996	0	1.142281	0.687671	0.377781	0	1.207558	0	0.942368	0	0.591904	1.891877
2.174368	0	1.195253	0.888889	0	0	0	0.655132	0.421023	0.275174	3.468884	1.804234	1.211438	0	0.80439	0.127291	0.137491
0	1.033435	0	3.518519	20.77151	26.89012	2.162983	0	1.375342	0.433748	0.487203	2.585595	8.867527	4.4081	0	1.190173	0
0	2.240556	1.335541	4.444444	3.219585	2.252218	1.081491	1.411053	0.684163	1.179982	1.864363	3.153857	8.475158	4.228972	1.699339	3.125	0
0	0	1.175612	0.6	0	0	0	0	0.88064	0.54102	0	0.781361	0	0	1.619433	0.470978	0.181488
0	0.312636	0.131871	0.281481	0	0	1.517883	0	0	0	0	0	0	0	0	0.076375	0
0	1.146331	1.374821	1.488889	0	0	5.492837	0	0	0	0.34429	0	3.565648	3.964174	0	5.180754	0
0	0	0	0	0.400593	0	0	0	0	0	0	0	0	0	0	0.260947	0
0	0	0.050504	0	1.617211	0.432244	0	0	0.157884	0	0.090945	0.568263	0	0.443925	0	0	0
0	0	0	0	0	0	0	0	0	0	0	0	0	0	0	0	0
0.648129	0	0.140288	0	0	0	0	0	0	0	0	0	0	0	0	0.439155	0
0	0	0	0	0	0	4.050849	0	0	0	1.727946	0.710328	0	0	0	0	0
11.24817	13.2436	0	0	0	0	1.897353	0	40.70942	14.32303	25.06821	14.91689	1.741135	6.970405	29.05924	0.280041	3.602266
19.8411	1.128962	54.93673	13.6963	0	0	0	0.9631	1.40341	4.328156	0.883461	3.580054	0	0	4.021948	29.60158	24.6824
0	0	0	0	0	0	2.295797	0	0.743807	0	0	0	0	0	0	0	0
0	39.49631	6.517774	0	1.275964	0.614241	1.55583	1.355059	11.16764	15.37242	21.82019	1.676374	0	0.521807	0.878969	2.743126	1.413408
0	0	0.446115	0	0	0	0	0	0.175426	0	0	0	0	0	0	0.076375	0
2.717959	0.790274	6.074465	6.066667	0	0	12.72175	0	6.950389	16.50576	0	0.895013	18.65712	10.49065	1.598125	7.071029	2.634329
0	0	0	0	0	0	4.572621	0	0	0	0	0	0	0	0	0	0
1.505331	0	0.429281	0.148148	0	0	0	0	0.073679	0.419757	0	0	0	0	0	0.598269	0
0	0	0	0	0	0	2.020681	1.276667	0	0.643627	0	0	0	0.607477	0.442148	0	0

TOC897.T3	TOC898.T0	TOC898.T3	TOC899.T3	TOC953.T0	TOC953.T3	TOC972.T0	TOC972.T3	TOC992.T0	TOC998.T0	TOC998.T3	TOC.CTRL1	TOC.CTRL1	TOC.CTRL1	TOC.CTRL1	TOC.CTRL1
0	0	0	2.569908	16.76898	3.267828	9.610998	14.7075	9.498636	3.423438	1.159651	4.187029	0	0	0	0
7.767505	0.513644	1.332329	0.973622	6.25525	3.759119	12.77243	8.467786	2.553718	6.539333	0.832116	13.07621	1.733891	8.301777	0.948429	1.57585
0	0	0	0	0	0	0	2.101855	0.443383	0	0	2.126535	0	0	0	3.594709
0	9.88764	75.75637	1.584965	23.56704	6.044365	4.840345	0.790254	4.165246	0	0	0	3.111166	0	0	0
0	0	0	0	0.084006	0	0	0.002744	0.017053	0	0	0	0	0	0	0
2.422229	0	2.121416	1.788747	1.479806	1.689742	0	0	1.027456	0	0	0	0	4.058646	0	6.695779
28.82115	35.92295	0	1.34156	4.807754	3.528361	2.768626	7.875096	67.5307	5.074458	0	1.968036	0	20.74959	0	0
0.202254	0	1.883501	0	0	0	0.399138	0.16738	0	0	0	0.198124	0	1.738033	0	3.417505
0	0	0	0	0.407108	0	0	0	0	0	0	0	0	0	0	0
0	0.82397	0	0	0.568659	0	0	0	0	0.048559	1.133094	0	0	0	0	0
0	0	0	0.883052	4.568659	1.578085	6.538267	4.939085	0.486016	3.156361	0.840968	2.826575	0	0.339839	0.484094	0
0	0	0	0.633986	1.667205	0.781599	1.843639	0.477445	0	1.108773	0.208029	0	0	0	1.481921	0
0	0	0	0	0	0	0.766599	0	0.162005	0	0.234586	0	0	0	0.731081	0
3.496099	0	3.703557	0	1.382876	0.878368	0	0.389639	0.366644	0.84979	0	1.611412	0.725529	0.407807	0	0
0	0	0	0.237745	0	0	0	0	0.869714	0.161865	0	0	0	0	0	0
0	0	2.347436	3.06238	0	1.124014	0.753928	0.389639	0	0	0	0	1.045253	0	0	0
1.073871	0	0.634442	0	0.878837	0	0.886974	0	0.127899	0	0.305404	0	0	1.679775	0.839755	0
16.18511	0	0	0	0.187399	0	0.266092	0.504884	0	0.922629	1.018014	2.773742	0	4.447034	0.059277	0
0	0	1.617828	0.701913	4.148627	2.13637	1.43183	2.090879	1.662688	3.067336	0	0	0	0	0.306264	0
0	0	0	1.330239	1.764136	1.637636	1.735935	4.991219	0.797237	4.224668	1.372106	0	0	0.582581	1.926497	0
2.167004	0.535045	0	0	0.504039	0	0.335783	0	0	0.420848	1.456203	1.334038	0	2.408001	0	0
0	0	0	0.203781	0.226171	0	0.234415	0	0.422067	0.412755	0	0	2.914412	0	0.592768	0
0	0.856073	0	0.424544	0	0	2.147745	0.565251	1.581685	5.147297	4.169433	0.647206	0	0.553452	0.849634	0
0	0	0	0.175478	0	0.580616	0	0	0	0.178051	0.292126	0	0	0	0	0
0	0	0	0	0.239095	0	0.253421	0.101526	0	0.194238	0	0	0	0	0	0
0	0	0	0	0	0.915587	0	0	0	0	0	0	0	0	0	0
0	0.160514	0	0	0.135703	0	0	0	0	0	0.017705	0	0	0	0	0
0.784937	0	0	0.356617	0.226171	0	0.386467	0	0.187585	0	0	0	0.221348	0	0	0
0.505634	1.861958	2.315714	45.56776	3.33441	16.95697	2.261784	5.496104	0	16.6235	0	1.651037	32.48893	3.777066	0.681683	0
24.13561	20.31033	0	0	2.210016	0.952806	2.325139	1.171661	1.513472	5.365814	39.71584	21.55594	0.897688	25.31314	11.9344	0
0	0.684858	0	0.005661	0	0	0	0	0	0	0	0	0	0	0	0
1.74805	2.343499	0.154645	16.29118	0.135703	4.95757	1.165737	2.088135	0	0	5.776125	0.70004	0	0.174774	1.985774	8.258971
0	0	0	0	0	0	0	0	0	0	0.694906	0	0	0	0	0
2.325917	0.963082	2.220548	1.500057	1.660743	1.317552	11.54334	19.14718	2.997101	13.07867	18.3641	5.124818	0.418101	8.903777	11.87512	0
0	0	0	0	6.326333	23.90948	0	0	0.251535	0	0	0	0	0	0	0
0	0	0	0	0.187399	0	0	0	0	0.008093	0.314257	0	0	0	0	0
0	0	0	0	0	0	0	0	0	0	0	4.702153	0	0	0	0

TOC.CTRL1	TOC.CTRL1	TOC.CTRL1	TOC.CTRL1	TOC.CTRL1	TOC.CTRL1	TOC.CTRL1	TOC.CTRL1	TOC.CTRL1	TOC.CTRL1	TOC.CTRL1	TOC.CTRL1	TOC.CTRL1	TOC.CTRL1	TOC.CTRL1	TOC.CTRL1	TOC.CTRL1
4.941154	0.995938	0	0	0.631295	0.491333	0	2.403418	6.987816	8.164629	3.176306	0	0	0	0	0	0.930959
21.78776	1.952562	0	0.98644	1.049794	8.55739	2.427184	2.492434	8.77108	33.68843	1.373538	0	0.784221	0.720279	2.45191	5.511274	0.856211
0	0.301402	34.65569	0	0	0	0	0	0	0	0	3.985171	0	0	0	0	0
0	0	0	0.451099	0.163144	1.815204	0	0.169129	22.00436	11.25437	0	0	0	0	8.00596	5.836392	3.961674
0	0	0	0	0.049652	0.006824	0	0.048959	0	0	0	0	0	0	0	0.015732	0
0	1.225265	0	0	0	0	0.910194	0.449528	4.454127	0.790125	0	0	1.235741	0	1.449472	0	0
0	0	0	0.494579	9.909207	2.36113	2.017597	12.92505	25.54668	2.95216	0	0	0	2.65949	1.910051	1.683272	1.692036
0	0.491417	6.287425	0	0	0	0	0	0	0	0	0	0	0	0	0	0
0	0	0	0	0	0	0	0	0.040345	0	0	0	0	0	0	0	0
0	0	0	0	0	0	0	0	0	0	0	0	0	0	0	0	0
2.100963	0.432447	0	0.125003	0.638388	1.194213	0.439927	1.513263	0.112967	2.279964	3.251422	2.224282	0.903042	0	3.982661	1.929733	0.142702
0	0.64867	0	0	0	0	0	0	0	0	0	0	0	0	1.828773	1.882538	0
0.894855	0	0	0	0	0.914426	0	0.778886	0	0	0	0	1.164449	0	0	0	0
1.400642	0	0	0.209245	1.071074	1.003139	0.424757	0.333808	1.452433	0	0	0	0.712928	0	0	1.672784	1.26393
0	0	0	1.269056	1.319336	2.886584	0	0.498487	0	0.467786	3.047537	0	0	0	0	0.251704	0
0	0	0	0	0	0	0	0	0	0	0	0	0	0	1.219182	0	0
0.729501	1.434936	0	0.527188	0.383033	0.818889	0.970874	0.34271	0.274348	0.235858	0	0	0.285171	1.163527	0	0.27268	0.353357
0.243167	0	0	0.22555	0	0	1.881068	0.640912	2.009199	0.4442	0	0	0.736692	0.300776	0	0.854746	4.688774
0	0	0	0.108699	4.922684	1.938037	0	0.930212	0.338901	0.797987	3.841614	0	0.760456	0	1.314007	4.472994	0
0.340434	0	0	0.233702	4.369414	3.261908	0.591626	0.97472	0.17752	1.073155	2.199807	0	0.617871	0	2.343538	6.224436	0.142702
2.13987	0	0	2.646811	0.581643	0.928074	0.879854	0.956917	0.282417	0	0.311192	0	0	0	0	0	3.832563
0.272347	0	0	0.057067	0.418499	0.225194	0	0	0	0.117929	0.4185	0	0	0	0	0.393288	0
1.682716	0	0	0.084241	3.362179	5.882353	0	0.86345	0	0.719368	4.657152	3.243744	0	0.174133	0.948253	7.661248	1.603697
0	0	0	0	0.482338	0.088713	0.515777	0.155777	0.60518	0	1.137461	0	0	0	0	0	0
0	0	0	0	0.205703	0	0	0	0	0.12186	0	0	0	0	0	0	0
0	0	0	0.595125	0	0	0	0	0	0	0	0	0	0	0	0.099633	0.543626
0	0	0	0	0.191517	0	0	0.324907	0	0	0	0	0	0	0	0	0
0	0	0	0	0	0	0	0	0	0	0	0	1.663498	0	0	0	0
0	15.57463	0	46.7866	0.716414	0	7.114684	1.891579	2.969418	4.174692	0	5.282669	2.685361	63.92275	12.31374	8.143681	0.448491
24.22916	0	0	2.679421	4.638956	5.861881	6.158981	31.21773	2.299685	1.281497	1.030153	21.59407	19.0827	0.7836	1.137903	1.782905	33.47377
0	0	0	0.595125	0	0	0	0	0	0	0	0	0	0	0	0	0
4.882794	23.06382	0	26.33495	1.773301	3.023065	20.41869	3.898878	8.504801	0.927709	0	0	2.091255	0	3.914928	3.801783	14.9701
0.106993	0	0	0	0	0	0.04551	0.324907	0	0	0	0	0	0	0	0	0.122316
3.180624	0.23588	0	1.054376	33.8204	32.51672	5.749393	11.62987	1.516985	21.10539	54.42644	12.32623	2.756654	0.862751	1.395286	22.60619	1.746398
0	0	0	0	0	0.388972	9.375	0	0	0	0	0	0	0	0	0	0
0	0	0	0	0.248262	0.272963	0	0.396119	0	0	0.096577	0	0	0	0.284476	0.13634	0.224246
0	0.687983	0	0	0	0	0	0	0	0	0	0	0	0	0	0	0

TOC.CTRL1	TOC.CTRL1	TOC.CTRL1	TOC.CTRL1	TOC.CTRL1	TOC.CTRL9	TOC.CTRL9	TOC.CTRL9	TOC.CTRL9	TOC.CTRL9	LEFSE HIGHLIGHTED
1.064626	0	1.597185	3.558467	0	0.54805	0	0	0	0.887269	Bacteria | Actinobacteria | Actinobacteria | Actinomycetales | Actinomycetace
5.726524	1.444891	1.691933	4.337435	4.586367	2.714759	3.972904	3.1618	41.91153	5.337368	Bacteria | Actinobacteria | Actinobacteria | Actinomycetales | Actinomycetace
0	0	0.44667	0	0	0	1.757598	0.086035	7.622433	0.453951	Bacteria | Actinobacteria | Actinobacteria | Corynebacteriales | Corynebacteri
0.1913	0	3.329724	2.171668	5.086974	0.369615	6.041743	3.769425	2.014218	0.832244	Bacteria | Actinobacteria | Actinobacteria | Micrococcales | Micrococcaceae |
0.024952	0	0	0	0.020227	0	0	0	0	0	Bacteria | Actinobacteria | Actinobacteria | Micrococcales | unclassified.F2 | u
0.677867	0.45381	0	0.607831	0	0	0	6.022477	0	2.96444	Bacteria | Actinobacteria | Actinobacteria | Micrococcales | Micrococcaceae |
75.32646	0.45381	4.507309	3.552566	0.581513	3.83635	16.97181	0	20.07899	5.743174	Bacteria | Actinobacteria | Actinobacteria | Micrococcales | Micrococcaceae |
0	0	0	0	0	0	0.823874	0.086035	5.837283	0.653415	Bacteria | Actinobacteria | Actinobacteria | Propionibacteriales | Propionibact
0	0	0	0	0.126416	0	0	0	0	0	Bacteria | Actinobacteria | Actinobacteria | unclassified.O2 | unclassified.F3 |
0	0	0	0	0	0	0	0	0	0	Bacteria | Actinobacteria | Coriobacteriia | Coriobacteriales | Atopobiaceae | /
0.523996	1.710918	0	0.548818	0.758495	0.344124	9.227389	1.064688	0.726698	1.485659	Bacteria | Actinobacteria | Coriobacteriia | Coriobacteriales | Atopobiaceae | /
0	0	0	0	0	0	0	0	0	0	Bacteria | Firmicutes | Clostridia | Clostridiales | Lachnospiraceae | Oribacteri
0	0.34427	0.649702	0.171137	1.08212	0	0	0	0	0.502098	Bacteria | Firmicutes | Clostridia | Clostridiales | Lachnospiraceae | Oribacteri
1.40148	0	1.218192	0.737659	2.270429	1.376498	0	1.935796	0	1.719513	Bacteria | Firmicutes | Clostridia | Clostridiales | Lachnospiraceae | Oribacteri
0	0.761567	0.44667	0.528164	2.523261	0	0	0.957144	0	0	Bacteria | Firmicutes | Clostridia | Clostridiales | Lachnospiraceae | Oribacteri
0	3.515727	0	0	0	0	0	0	0	0	Bacteria | Firmicutes | Clostridia | Clostridiales | Lachnospiraceae | Catonella |
0	0	0.500812	0.702251	0.854571	1.147081	0	0	0	0.282	Bacteria | Firmicutes | Clostridia | Clostridiales | Lachnospiraceae | Catonella |
2.254013	0	2.17921	1.183205	2.533374	1.816212	0	0.284992	0	1.526928	Bacteria | Firmicutes | Clostridia | Clostridiales | Lachnospiraceae | Stomatoba
0	1.919566	0	1.348441	0.834345	0	0	0	0	0	Bacteria | Firmicutes | Clostridia | Clostridiales | Lachnospiraceae | Stomatoba
0	1.434458	0.406064	1.510726	1.183252	0	0	0	0.774092	0	Bacteria | Firmicutes | Clostridia | Clostridiales | Lachnospiraceae | Lachnoana
0.752724	0.125189	1.746075	1.493022	1.790049	2.848585	0.512633	0.69366	0	0.921659	Bacteria | Firmicutes | Clostridia | Clostridiales | Lachnospiraceae | Lachnoana
0.06238	0.104324	0	0.817326	0.091019	0	0	1.661558	0	0	Bacteria | Firmicutes | Clostridia | Clostridiales | Lachnospiraceae | Butyrivibri
0	0.83981	2.138603	1.062229	0.940534	3.76625	0.988649	0	2.622433	0.275122	Bacteria | Firmicutes | Clostridia | Clostridiales | Lachnospiraceae | Johnsonell
0	0.380783	0	0	0.055623	0	0	0	0	0	Bacteria | Firmicutes | Clostridia | Clostridiales | Lachnospiraceae | unclassifie
0	0.177351	0	0	0	0	0.201391	0	0	0	Bacteria | Firmicutes | Clostridia | Clostridiales | Family_XIII | Mogibacterium
0.141396	0.172135	0.135355	0	0.055623	1.994647	0	0	0	0	Bacteria | Firmicutes | Clostridia | Clostridiales | Peptostreptococcaceae | unc
0	0	0	0.085568	0.091019	0	0	0	0	0	Bacteria | Bacteroidetes | Bacteroidia | Bacteroidales | unclassified.F4 | unclas
0	0.078243	0.568489	0	0	0	0	0	0	0	Bacteria | Fusobacteria | Fusobacteriia | Fusobacteriales | Leptotrichiaceae | L
0.25368	24.99609	12.39848	24.40766	0	0	7.506408	0	1.034755	0	Bacteria | Fusobacteria | Fusobacteriia | Fusobacteriales | Fusobacteriaceae |
2.765533	1.606593	10.34109	12.09466	37.38875	24.47744	3.936287	43.40485	4.099526	31.50836	Bacteria | Fusobacteria | Fusobacteriia | Fusobacteriales | Fusobacteriaceae |
0	0	0	0.442595	0	0	0	0	0	0	Bacteria | Fusobacteria | Fusobacteriia | Fusobacteriales | Fusobacteriaceae |
0.89412	0.015649	3.099621	15.3197	3.18568	16.93219	3.405346	5.699844	6.21643	12.91698	Bacteria | Fusobacteria | Fusobacteriia | Fusobacteriales | Fusobacteriaceae |
0	0	0	0.076717	0.121359	0	0	0	0	0	Bacteria | Fusobacteria | Fusobacteriia | Fusobacteriales | Fusobacteriaceae |
0.677867	2.336863	6.199242	6.836623	14.16363	14.3194	1.611131	14.31951	0.971564	8.920834	Bacteria | Fusobacteria | Fusobacteriia | Fusobacteriales | Leptotrichiaceae | L
0.336854	11.78864	0	0	0	0	10.8019	0	0	0	Bacteria | Fusobacteria | Fusobacteriia | Fusobacteriales | Leptotrichiaceae | (
0	0	0.406064	0.156384	0.293285	0	0	0	0	0	Bacteria | Fusobacteria | Fusobacteriia | Fusobacteriales | unclassified.F11 | u
0	0	0	0	0	0	0	0	0	0	Bacteria | Fusobacteria | Fusobacteriia | Fusobacteriales | Leptotrichiaceae | L

eae | Actinomyces | Actinomyces odontolyticus
eae | Actinomyces | Actinomyces unclassified.S2
aceae | Corynebacterium | Corynebacterium unclassified.S7
Rothia | Rothia unclassified.S18
nclassified.G3 | unclassified.S19
Rothia | Rothia dentocariosa
Rothia | Rothia mucilaginosa
eriaceae | Cutibacterium | Cutibacterium unclassified.S21
unclassified.G4 | unclassified.S22
Atopobium | Atopobium unclassified.S23
Atopobium | Atopobium parvulum
um | Oribacterium asaccharolyticum
um | Oribacterium parvum
um | Oribacterium sinus
um | Oribacterium unclassified.S81
| Catonella morbi
| Catonella unclassified.S73
aculum | Stomatobaculum unclassified.S83
aculum | Stomatobaculum longum
erobaculum | Lachnoanaerobaculum unclassified.S76
erobaculum | Lachnoanaerobaculum cf.
o | Butyrivibrio unclassified.S72
la | Johnsonella unclassified.S75
d.G21 | unclassified.S84
| Mogibacterium unclassified.S65
lassified.G22 | unclassified.S88
sified.G8 | unclassified.S37
eptotrichia | Leptotrichia hongkongensis
Fusobacterium | Fusobacterium nucleatum
Fusobacterium | Fusobacterium periodonticum
Fusobacterium | Fusobacterium simiae
Fusobacterium | Fusobacterium unclassified.S106
unclassified.G26 | unclassified.S107
eptotrichia | Leptotrichia unclassified.S108
Oceanivirga | Oceanivirga unclassified.S109
nclassified.G28 | unclassified.S113
eptotrichia | Leptotrichia wadei
